# Disruption of mitochondrial dynamics affects behaviour and lifespan in *Caenorhabditis elegans*

**DOI:** 10.1007/s00018-019-03024-5

**Published:** 2019-03-06

**Authors:** Joseph J. Byrne, Ming S. Soh, Gursimran Chandhok, Tarika Vijayaraghavan, Jean-Sébastien Teoh, Simon Crawford, Ansa E. Cobham, Nethmi M. B. Yapa, Christen K. Mirth, Brent Neumann

**Affiliations:** 1 Neuroscience Program, Monash Biomedicine Discovery Institute and Department of Anatomy and Developmental Biology, Monash University, Melbourne, VIC 3800 Australia; 2 Monash Ramaciotti Centre for Cryo-Electron Microscopy, Monash University, Melbourne, VIC 3800 Australia; 3 School of Biological Sciences, Monash University, Melbourne, VIC 3800 Australia

**Keywords:** Mitochondria, Mitochondrial dynamics, Mitofusin 1, Mitofusin 2, FZO-1, OPA1, EAT-3, DRP1, DRP-1, *Caenorhabditis elegans*, Transmission electron microscopy

## Abstract

Mitochondria are essential components of eukaryotic cells, carrying out critical physiological processes that include energy production and calcium buffering. Consequently, mitochondrial dysfunction is associated with a range of human diseases. Fundamental to their function is the ability to transition through fission and fusion states, which is regulated by several GTPases. Here, we have developed new methods for the non-subjective quantification of mitochondrial morphology in muscle and neuronal cells of *Caenorhabditis elegans*. Using these techniques, we uncover surprising tissue-specific differences in mitochondrial morphology when fusion or fission proteins are absent. From ultrastructural analysis, we reveal a novel role for the fusion protein FZO-1/mitofusin 2 in regulating the structure of the inner mitochondrial membrane. Moreover, we have determined the influence of the individual mitochondrial fission (DRP-1/DRP1) and fusion (FZO-1/mitofusin 1,2; EAT-3/OPA1) proteins on animal behaviour and lifespan. We show that loss of these mitochondrial fusion or fission regulators induced age-dependent and progressive deficits in animal movement, as well as in muscle and neuronal function. Our results reveal that disruption of fusion induces more profound defects than lack of fission on animal behaviour and tissue function, and imply that while fusion is required throughout life, fission is more important later in life likely to combat ageing-associated stressors. Furthermore, our data demonstrate that mitochondrial function is not strictly dependent on morphology, with no correlation found between morphological changes and behavioural defects. Surprisingly, we find that disruption of either mitochondrial fission or fusion significantly reduces median lifespan, but maximal lifespan is unchanged, demonstrating that mitochondrial dynamics play an important role in limiting variance in longevity across isogenic populations. Overall, our study provides important new insights into the central role of mitochondrial dynamics in maintaining organismal health.

**Electronic supplementary material:**

The online version of this article (10.1007/s00018-019-03024-5) contains supplementary material, which is available to authorized users.

## Introduction

Mitochondria exist not just as isolated organelles, but also as highly interconnected networks, constantly shifting between these two states via a balance between opposing fusion and fission forces. This ability to transition between fission/fusion states is essential for the functions of mitochondria in maintaining optimal cellular bioenergetics, in regulating intracellular Ca^2+^, and in cellular stress responses. Fusion allows damaged mitochondria to mitigate stress by mixing their contents as a form of functional complementation [1], diluting accumulated mitochondrial DNA mutations and oxidised proteins. Fission contributes to quality control of damaged mitochondria by budding off deteriorating components for targeted breakdown via autophagy or mitophagy [2, 3]. Disruption of this homeostasis can have major consequences for mitochondrial health and function [4, 5], and thus influence larger processes such as behaviour and neuronal circuitry [6]. Despite this link, our understanding of the role of mitochondrial dynamics in regulating animal behaviour and lifespan remains incomplete.

In humans, mitofusin 1 and 2 orchestrate mitochondrial fusion together with optic atrophy-1 (OPA1). Mitofusin proteins form dimeric interactions across outer mitochondrial membranes, tethering adjacent mitochondria and inducing fusion through GTP hydrolysis [7]. The dynamin-like GTPase OPA1 is essential for fusion of the inner mitochondrial membrane [8], as well as maintenance of the internal cristae structure [9]. Mitochondrial fission is mediated by another member of the dynamin family, dynamin-related protein 1 (DRP1), which upon activation is recruited from the cytosol with the help of mitochondrial fission factor (Mff) and mitochondrial dynamics proteins (MiD49 and MiD51) to form a spiral around mitochondria that constricts to sever both inner and outer membranes [10, 11]. Malfunction of mitochondrial dynamics has been linked to a number of diseases, in particular diseases with neuronal and skeletal muscle manifestations. More than 100 mutations within the gene encoding mitofusin 2 (*MFN2)* have been attributed to subtype 2A of Charcot–Marie–Tooth disease, the most common heritable axonal neuropathy [7, 12]. OPA1 was originally discovered in autosomal dominant optic atrophy, the most common form of hereditary optic neuropathy, for which around 250 pathogenic mutations have been described [13, 14]. Mutation of DRP1 causes a severe form of infantile neurodegenerative disease [15], and over-activation of DRP1-mediated fission is associated with a number of diseases, including Parkinson’s and Huntington’s diseases [16]. Understanding how disruption of mitochondrial dynamics affects health in the context of a whole organism would therefore be beneficial in deciphering the biological role of fission/fusion machinery in disease.

Individual knockouts of *MFN1/2*, *OPA1* or *DRP1* are embryonically lethal in mice [17–20]; however, knockouts in the nematode *Caenorhabditis elegans* are viable and have therefore allowed us to dissect the roles of the individual mitochondrial fission and fusion proteins in organismal health. In *C. elegans*, FZO-1 is the sole mitofusin orthologue [21], EAT-3 is the orthologue of OPA1 [4], and the orthologue of DRP1 is DRP-1 [22]. Previous studies have largely relied upon subjective, categorical measurements to quantify changes in mitochondrial morphology when these proteins are disrupted. In this study, we have developed non-subjective methods to assess mitochondrial morphology, allowing us to identify surprising tissue-specific differences when fusion or fission proteins are disrupted. Using transmission electron microscopy, we reveal a novel function for FZO-1 in mediating the structure of the inner mitochondria membrane, with severe cristae disruption evident when this protein is absent. Furthermore, we reveal the impact of loss of fission/fusion machinery through detailed analysis of tissue-specific behavioural paradigms and animal lifespan, highlighting the vital importance of mitochondrial dynamics for organismal fitness. Our results reveal that disruption of the fission/fusion balance in either direction results in similar consequences, although disruption of fusion is in most instances more profound. Intriguingly, our data also provide evidence supporting a role for mitochondrial dynamics in limiting variability in lifespan across a population.

## Results

### Knockout of the fission/fusion proteins disrupts mitochondrial morphology in muscles

**Fig. 1:**
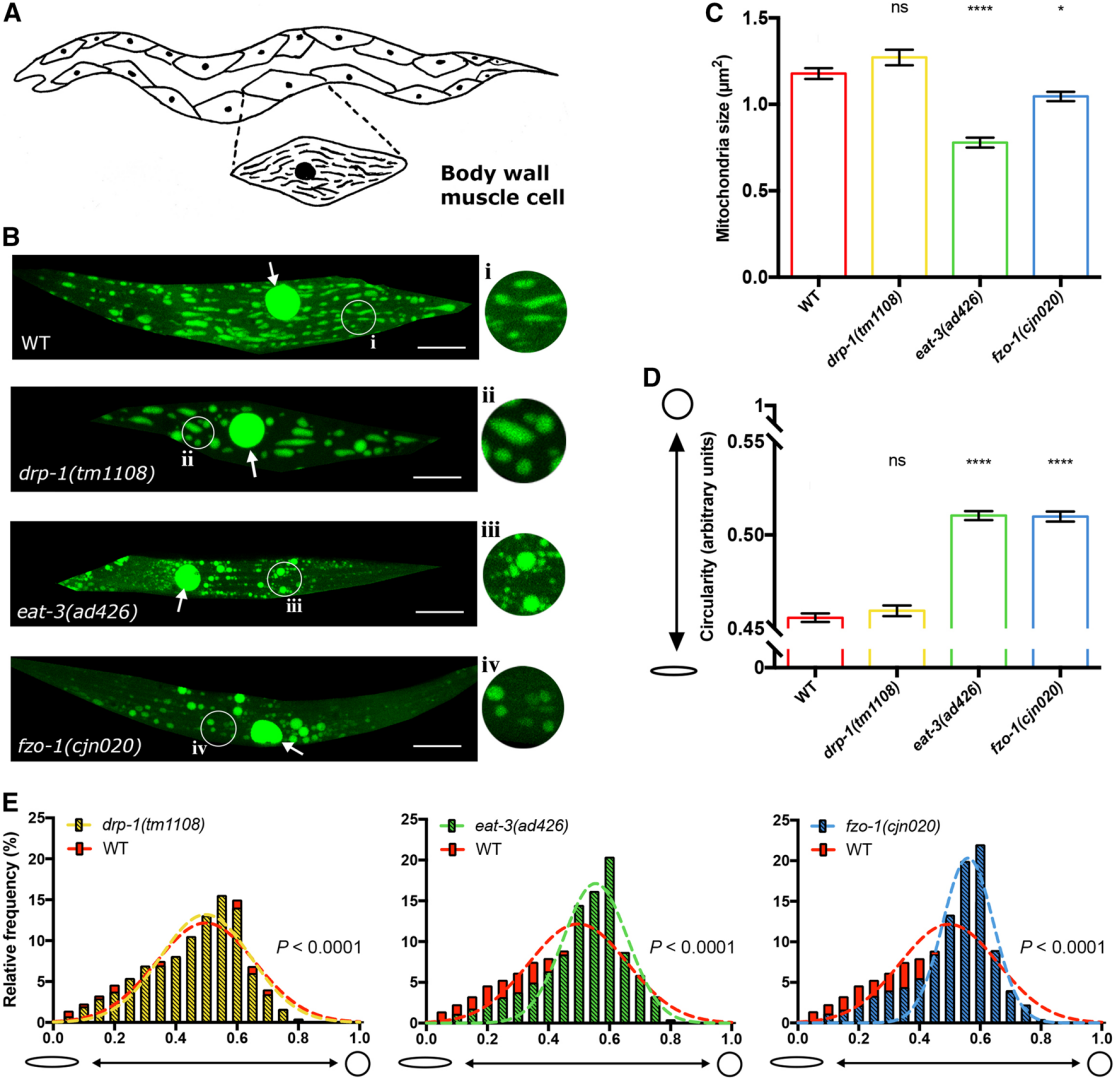
Mitochondrial morphology in body wall muscles. **a** Schematic of the body wall muscle cells of *C. elegans,* which are arranged in four longitudinal bundles comprised 95 cells. Inset shows the mitochondrial network within a single muscle cell. **b** Representative images of body wall muscle with fluorescently labelled mitochondria (*Pmyo*-*3::MLS::GFP*). Arrows designate nuclei, which are also labelled with GFP in this transgenic strain. *drp*-*1(tm1108)* animals show elongated mitochondria similar to wild type as seen more closely in insets **i** and **ii**. *eat*-*3(ad426)* and *fzo*-*1(cjn020)* mutants exhibit more fragmented mitochondrial networks compared to wild type (**iii**, **iv** compared with **i**). Images are representative of  ≥ 10 muscle cells from nine worms analysed per genotype, visualised via confocal microscopy. Scale bars 20 μm. Insets are enlargements (× 3) of the indicated circular areas. **c** Mean size (µm^2^) of mitochondria in the body wall muscles as determined using object segmentation (SQUASSH). **d** Mean circularity of mitochondria in the body wall muscles, calculated by fitting each object to a perfect circle and measuring deviation using the following formula (4 × *π*) × (Area/Perimeter^2^). A value of 1 represents a perfect circle, and 0 a straight line. Histogram and Gaussian distribution of mitochondria circularity scores for each genotype. All three variances were statistically different (*P* < 0.0001) when compared to wild type (*F* test for variances). All imaging was performed on L4 animals. Data are represented as mean ± SEM. *P < 0.05, ****P < 0.0001 from one-way ANOVA with Dunnett’s post hoc tests for multiple comparisons; *n* ≥ 2525 mitochondria for quantitative analysis

To elucidate how mitochondrial dynamics are disrupted in the absence of mitochondrial fission/fusion proteins, we first performed detailed analyses in muscle cells in animals lacking either DRP-1, EAT-3 or FZO-1. Fusion and fission dynamics influence the maintenance of organelle homeostasis, and disruption of these dynamics has previously been shown to alter mitochondrial morphology in a range of different organisms, including *C. elegans* [4, 5, 23–25]. *C. elegans* lacking either of the three fission/fusion proteins are viable, allowing us to use strong loss-of-function alleles or null mutations. For *drp*-*1* we used the *tm1108* allele that causes a 407 bp deletion within the dynamin GTPase domain [22], and for *eat*-*3* we used the *ad426* allele, a loss-of-function allele inducing a V328I substitution within the dynamin domain. Due to an uncharacterised background mutation in the available *fzo*-*1(tm1133)* strain that induces morphological defects in the nervous system that are not related to the mutation of *fzo*-*1* (Byrne and Neumann, unpublished observations), we used a CRISPR/Cas-9 approach to generate a new, null allele of *fzo*-*1*, *cjn020*. We examined the morphology and distribution of fluorescently labelled mitochondria in these mutant backgrounds, using differences in mitochondrial size and shape compared to wild type animals as a readout for changes in mitochondrial dynamics. Mitochondria within the body wall muscles were analysed using a *Pmyo3::mitochondrial*-*GFP* transgene in larval stage 4 (L4) animals (Fig. 1). We quantified mitochondrial size and shape using the SQUASSH segmentation ImageJ macro [26] (Supplementary Fig. 1A; Supplementary Table 1). Animals lacking either EAT-3 or FZO-1 displayed a significant decrease in mitochondrial size compared to wild type (34% and 11% reductions, respectively), whereas *drp*-*1* mutants presented no significant changes (Fig. 1a–c, Supplementary Fig. 1B). To measure shape changes we analysed circularity. Both the *eat*-*3(ad426)* and *fzo*-*1(cjn020)* fusion mutants showed significantly increased circularity (Fig. 1d). Surprisingly, despite the presence of more elongated mitochondria in some muscle cells, *drp*-*1(tm1108)* mutants again showed no significant difference compared to the wild type (Fig. 1d, Supplementary Fig. 1B). To study this more closely, we plotted the frequency of circularity scores and fitted a Gaussian distribution for each genotype. This analysis revealed significant differences for all three genotypes compared to the variance in wild type animals (Fig. 1e). Overall and as expected, our data indicate that mitochondria in the fusion mutants show a more fragmented arrangement, exhibiting a decrease in size and increase in circular shape, as previously reported [4, 5]. However, our data do not show the hyper-connected phenotype previously observed with RNAi knockdown of *drp*-*1* [21, 24]. Instead, our findings support those of Ackema et al. who demonstrated differences in mitochondrial morphology when *drp*-*1* was knocked down versus knocked out, with blebs rather than hyper-connection the predominant phenotype in animals carrying the *drp*-*1(tm1108)* null allele [27, 28]. This blebbing phenotype has been proposed to result from fission of the inner mitochondrial membrane still occurring in the absence of DRP-1 [24, 28].

### Ultrastructural analysis of mitochondria

**Fig. 2:**
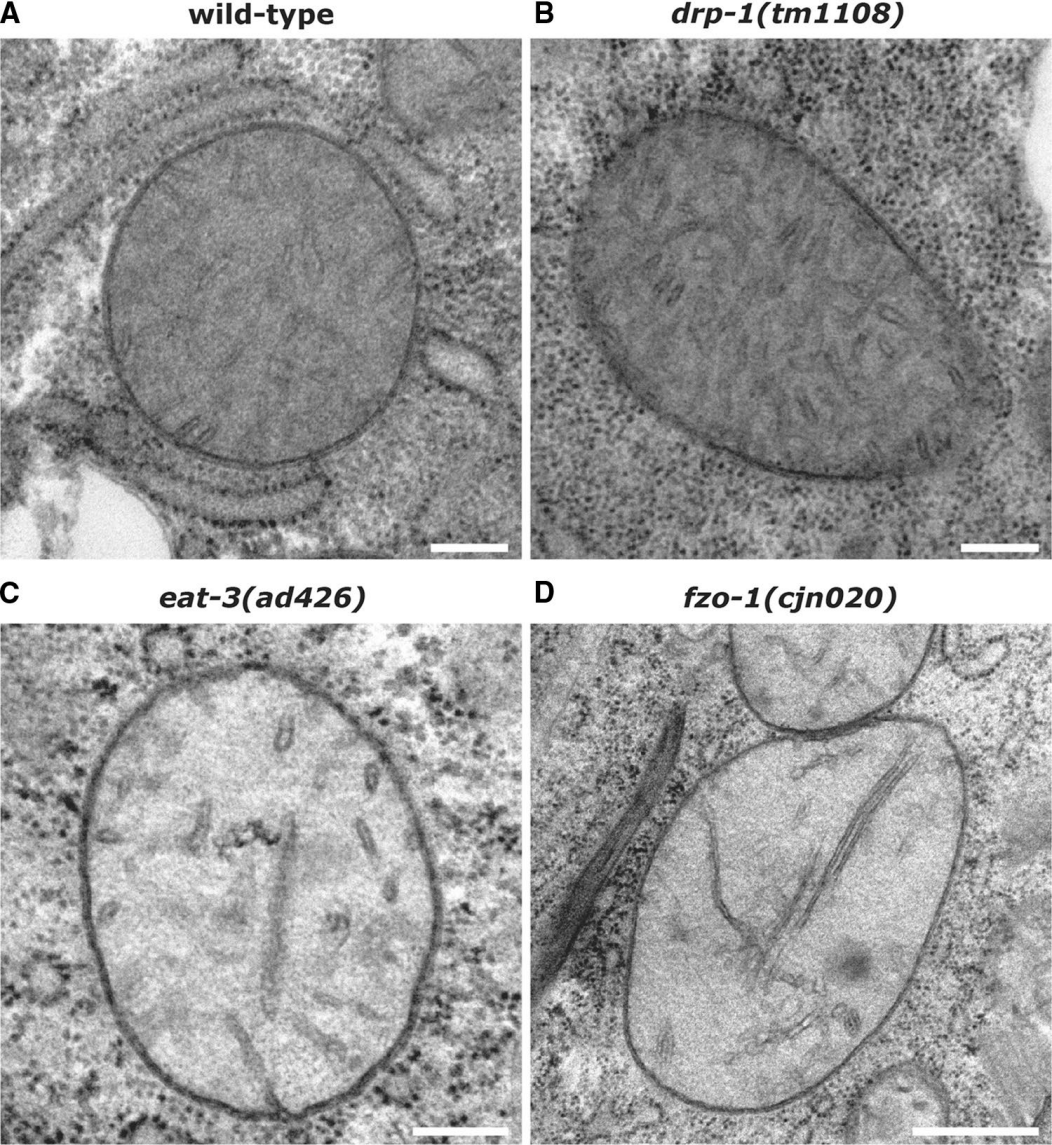
Ultrastructural analysis of mitochondria. Transverse sections captured with electron microscopy of mitochondria from the body wall muscles of L4 stage; **a** wild type, **b***drp*-*1(tm1108)*, **c***eat*-*3(ad426)*, and **d***fzo*-*1(cjn020)* mutant animals. Images representative of a total of 39 mitochondria imaged for wild type, 31 for *drp*-*1(tm1108)*, 34 for *eat*-*3(ad426)*, and 51 for *fzo*-*1(cjn020)*. Further examples for each genotype are displayed in Supplementary Fig. 2. Scale bars 200 nm

To further scrutinise mitochondrial morphology in animals lacking the fission and fusion genes, we performed electron microscopy analyses. In line with previous studies [4, 29], mitochondria in the body wall muscles of wild type animals displayed highly consistent appearances, with regular outer membrane shape and dense cristae throughout each organelle (Fig. 2a, Supplementary Fig. 2A). Mitochondria in animals lacking *drp*-*1* were similar to wild type in appearance (Fig. 2b). Despite the presence of some enlarged mitochondria in these animals, membrane structures all appeared normal (Supplementary Fig. 2B). In contrast, mitochondria in *eat*-*3* and *fzo*-*1* mutants were mostly spherical and frequently dysmorphic. EAT-3 and its mammalian orthologue OPA1 are required for maintaining mitochondrial cristae [22, 30]. As such, loss of *eat*-*3* disrupted cristae structure, with reduced density and apparently shorter cristae (Fig. 2c, Supplementary Fig. 2C). In addition, 12% of the mitochondria observed displayed inner membrane septae (4/34) and 15% had electron-dense inclusions (5/34; Supplementary Fig. 2C). Unexpectedly, loss of FZO-1 had a similar effect on mitochondrial morphology as loss of EAT-3 (Fig. 2d, Supplementary Fig. 2D). To our knowledge, FZO-1/mitofusin 2 has not previously been implicated in modulating cristae structure. Although the cristae in some *fzo*-*1* mutant mitochondria appeared as per wild type, the majority appeared less dense and either short or in many cases elongated and narrow. Electron-dense inclusions were also observed in 22% of *fzo*-*1* mutant mitochondria (11/51), as were inclusion bodies in 10% of those analysed (5/51; Supplementary Fig. 2D). The aberrant cristae in FZO-1-deficient animals may suggest a role for FZO-1 in cristae formation or maintenance. Overall, our ultrastructural analyses support and significantly extend our findings on mitochondrial morphology, revealing substantial internal structural disruptions when the fusion machinery is absent.

### Disruption of mitochondrial dynamics affects animal behaviour

**Fig. 3:**
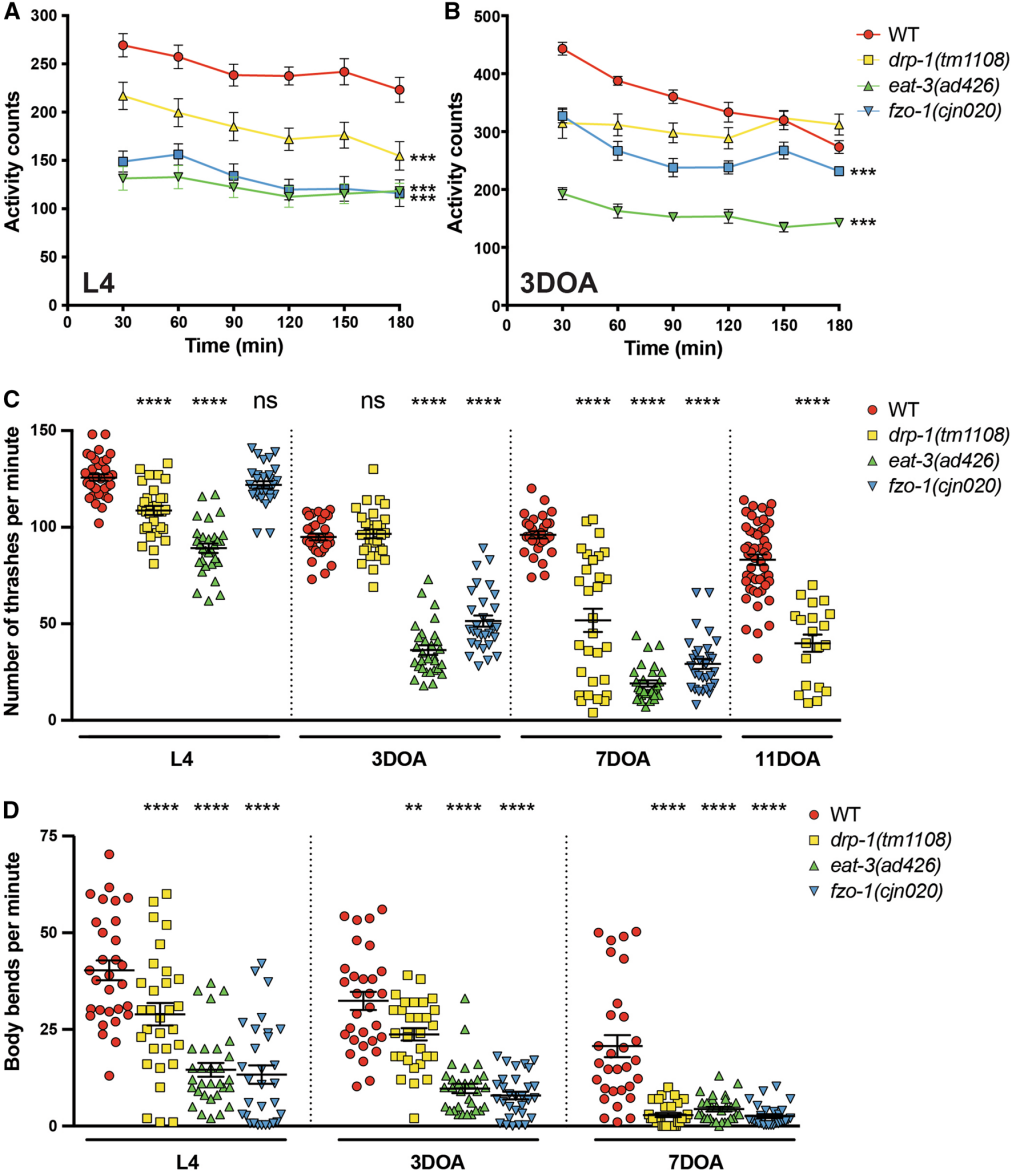
Disruption of mitochondrial dynamics affects animal movement. **a** Quantified movement across populations of L4-stage animals using a WMicrotracker instrument. The WMicrotracker records movement in multi-well plates using infrared beams. Data represent the mean ± SEM across 15 individual wells; *n* ≥ 900 worms per genotype. ***P < 0.001 compared to WT. **b** Quantification of the movement of populations of 3DOA animals using a WMicrotracker instrument. Data represent the mean ± SEM across 12 individual wells; *n* ≥ 360 worms per genotype. ***P < 0.001 compared to WT. **c** Number of thrashes per minute in liquid. At L4 stage *eat*-*3(ad426)* and *drp*-*1(tm1108)* show reduced thrashes per minute (29% and 13.6%, respectively). At 3DOA stage, the fusion mutants, *fzo*-*1(cjn020)* and *eat*-*3(ad426)*, show ≥ 60% reduction in thrashes per minute, and *drp*-*1(tm1108)* shows no significant reduction. Analysis of 7DOA and 11DOA animals reveals defects in *drp*-*1(tm1108)* animals (46% and 52%, respectively). Symbols represent individual animals over three replicate experiments; *n* ≥ 30 worms. **d** Quantification of the number of body bends for *drp*-*1(tm1108), eat*-*3(ad426)*, and *fzo*-*1(cjn020)* compared to WT. All three mutants show reduced sinusoidal bends per minute (on solid, not liquid media) across all ages tested. Symbols show individual animals from three replicates; *n* ≥ 30 worms. Data are represented as the mean, ± SEM. **P < 0.01, ****P < 0.0001 from one-way ANOVA with Dunnett’s post hoc tests for multiple comparisons

Given the morphological differences in mitochondria when dynamics are disrupted, we examined a number of specific behaviours to define how loss of the fusion/fission machinery affects muscle function. Firstly, we studied animal movement. To provide automated quantification of animal movement we used a WMicrotracker instrument [31], which records movement in multiwell plates using infrared beams. As shown in Fig. 3a, all three mitochondrial fission and fusion mutants displayed significant deficits in movement over a 3 h period compared to wild type animals at the L4 stage. Loss of either of the fusion genes led to a twofold reduction in animal movement, whereas loss of fission induced an intermediate phenotype. In adult animals (3-day-old adult, 3DOA), both fusion mutants again displayed severely reduced activity compared to the wild type, but *eat*-*3* mutants were now much reduced in movement compared to the *fzo*-*1* mutants. Interestingly, adult *drp*-*1* mutants displayed no significant difference to wild type animals (Fig. 3b). These data demonstrate that the overall activity of *C. elegans* is strongly impaired in the absence of the mitochondrial fusion and fission proteins. However, it is unclear why the fission protein DRP-1 is crucial for normal activity during development, but redundant in adult animals in this assay. This disparity may point towards underlying differences in the requirement for mitochondrial fission during development compared to adulthood.

To further define these differences and on an individual animal level, we analysed the swimming behaviour (also known as thrashing) of *C. elegans* [32]. We counted the number of completed thrashes per minute, and observed an age-dependent defect across all three mutant strains (Fig. 3c). At the L4 stage, the absence of DRP-1 or EAT-3 induced a reduced thrashing rate, whereas no defect was observed in animals lacking FZO-1 (Fig. 3c). In early adulthood (3DOA), mutation of either fusion gene caused an approximate 60% reduction in thrashes per minute, but surprisingly *drp*-*1* mutants were not significantly different to wild type animals (Fig. 3c). However, at later ages (7DOA and 11DOA) loss of DRP-1 induced significant reductions in thrashing rates (Fig. 3c). To ensure the movement defects we observed were due to mutations in the chosen genes and not background mutations, thrash assays were performed in additional alleles of each gene: *drp*-*1(or1393), eat*-*3(tm1107)* and *fzo*-*1(tm1133)* (Supplementary Fig. 3A, B). The *or1393* allele induces G39E missense mutations in the GTPase domain of DRP-1 [33], *eat*-*3(tm1107)* is a deletion of 417 bp in the dynamin domain, and *fzo*-*1(tm1133)* a 405 bp deletion within the dynamin/mitofusin GTPase domains [22]. The defective thrash phenotype was replicated in all three mutants, showing no significant difference between alleles of the same genes (Supplementary Fig. 3A, B). Thus, loss of mitochondrial dynamics significantly affects animal movement in an age-dependent and progressive nature.

To analyse locomotion, we quantified the characteristic sinusoidal bends of *C. elegans* [32]. Compared to wild type animals, *drp*-*1, eat*-*3* and *fzo*-*1* mutants all showed a reduction in body bends across both developmental (L4) and adult stages (Fig. 3d). Animals carrying the *eat*-*3* or *fzo*-*1* mutations were significantly reduced at L4, 3DOA and 7DOA, with the defect most striking at 3DOA (74% and 76% reductions compared to wild type, respectively). Animals lacking DRP-1 also had a significant reduction in body bends across all ages tested, albeit to a lesser extent at L4 and 3DOA stages. The defect in this background was most pronounced at 7DOA, with a 75% reduction in body bends compared to wild type. To ensure that reduced body bends were not a result of disrupted exploration behaviour, we assessed the average area covered by each mutant after 16 h [34, 35] and found no significant difference compared to wild type (Supplementary Fig. 3C). Overall, our movement analyses reveal the critical importance of mitochondrial dynamics proteins for normal activity of animals within a population (WMicrotracker analysis), and on an individual animal level (thrash and body bend assays).

### Muscle strength is disrupted by loss of mitochondrial fission and fusion

**Fig. 4:**
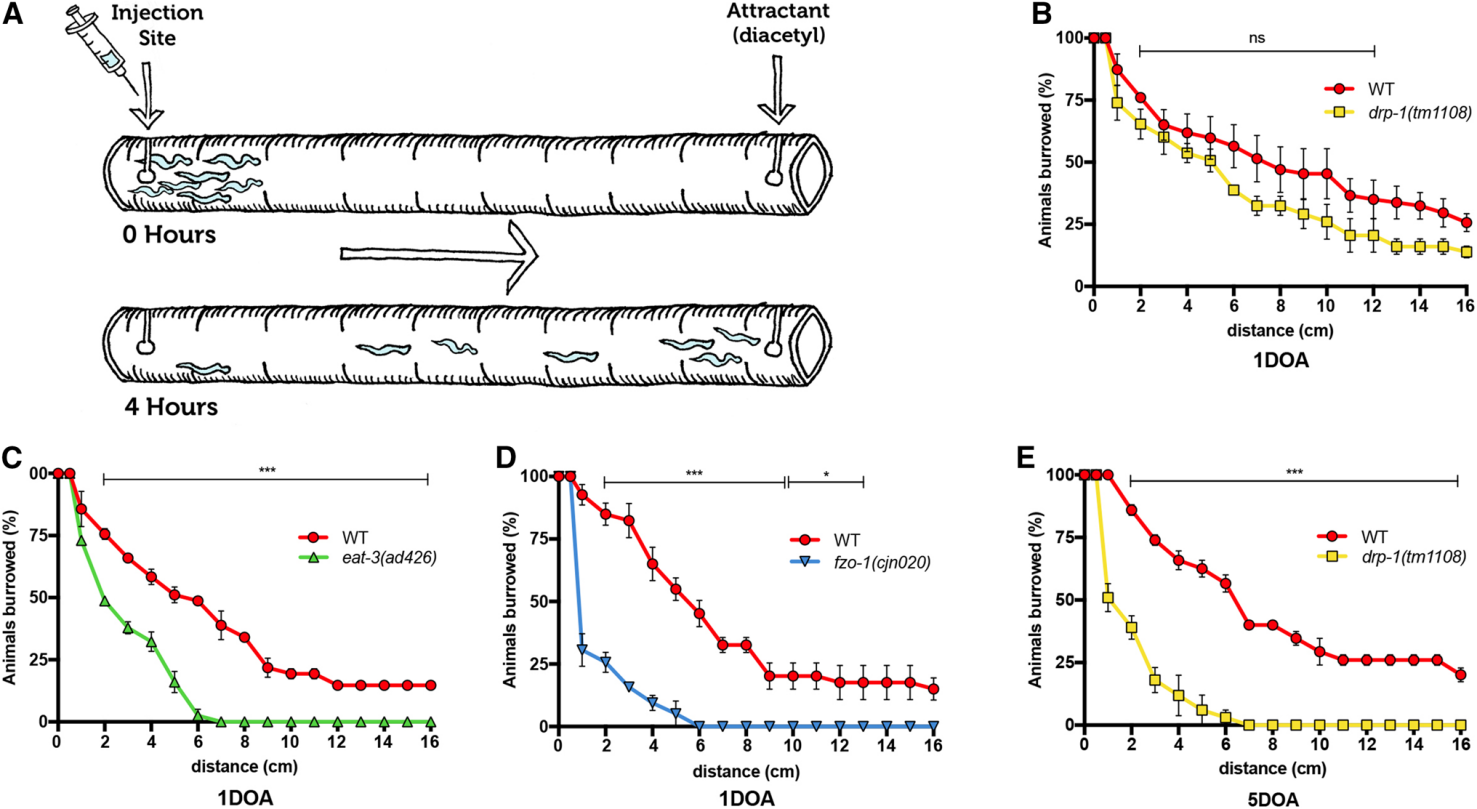
Muscle strength is disrupted by loss of mitochondrial fission and fusion. **a** Schematic representation of the burrowing assay used to quantify muscle strength in *C. elegans*. An agar-filled serological pipette is injected with worms at one end, and an attractant (diacetyl) at the other. Worms burrow through the agar towards the attractant and the distance (in cm) moved is measured for each animal after 4 h. **b** Burrowing assay for 1DOA *drp*-*1(tm1108)* worms show no significant difference in distance moved compared to wild type. **c***eat*-*3(ad426)* and **d***fzo*-*1(cjn020)* show significantly reduced distance burrowed at 1DOA compared to wild type. **e***drp*-*1(tm1108)* show significantly reduced distance burrowed at 5DOA. Data is represented as mean ± SEM cumulative distance covered across three replicate experiments. *n* ≥ 36 worms. ^ns^*P *> 0.05, *P < 0.05, ***P < 0.001 from multiple *t* tests per row using the Holm–Sidak method

Movement deficits in liquid and solid media suggest that muscle health is affected by the absence of mitochondrial dynamics proteins. To more specifically assess muscle strength, we challenged worms to burrow through different densities of agar towards an attractant odour (diacetyl), using a modified version of the protocol developed by Beron et al. [36]. After 4 h, animal positions relative to the starting point were measured, providing a quantitative readout for muscle strength (Fig. 4a). Chemotaxis assays were used to confirm that animals carrying mutations in *drp*-*1*, *eat*-*3* or *fzo*-*1* still displayed attraction towards diacetyl (Supplementary Fig. 3D). In 1.5% agar, 1DOA *drp*-*1* mutants showed no significant difference in distance burrowed compared to wild type (Fig. 4b). Strikingly, *eat*-*3* and *fzo*-*1* mutants displayed significantly reduced burrowing ability compared to the wild type (Fig. 4c, d), suggesting that muscle strength is already compromised after 1 day of adulthood in the fusion mutants. As a control, we compared the burrowing strength of *fzo*-*1* mutants to *dys*-*1(eg33)* animals, which lack the functional orthologue of the dystrophin protein that is implicated in Duchenne muscular dystrophy and previously shown to be highly defective in the burrowing assay [36]. The reduction in muscle strength in animals lacking FZO-1 was similar to that of animals carrying the *dys*-*1* mutation (Supplementary Fig. 3E). To ascertain whether loss of DRP-1 function would affect muscle strength at older ages, we analysed 5DOA animals. Animals lacking *drp*-*1* were severely affected at this age, showing similar defects to the younger fusion mutants (Fig. 4e). Therefore, mitochondrial dynamics are essential for muscle strength, and our data suggest that the absence of fusion is more detrimental earlier in life than the lack of fission.

### Disruption of fission/fusion proteins affects mitochondrial morphology in a tissue-specific manner

**Fig. 5:**
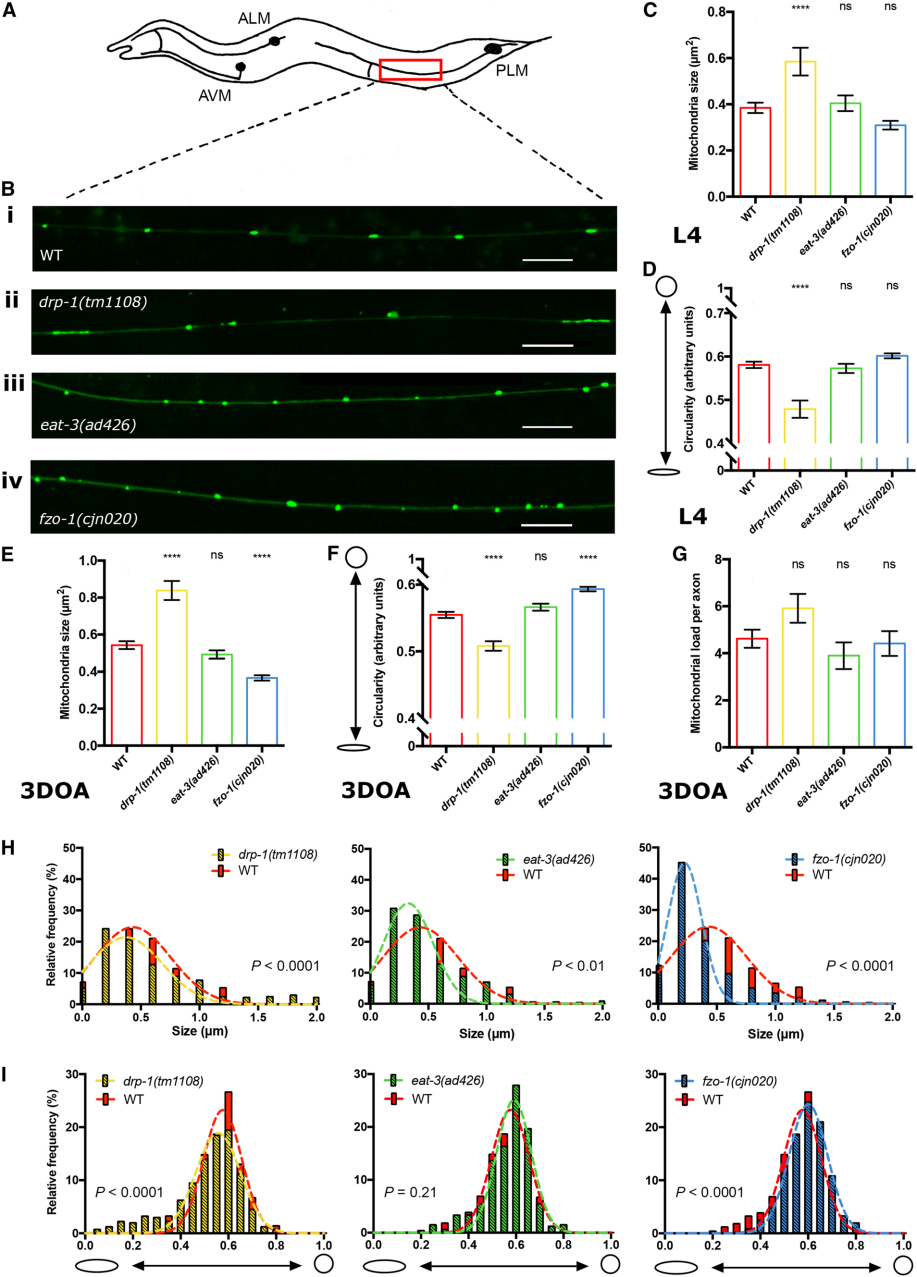
Mitochondrial morphology in PLM neurons. **a** Schematic of the *C. elegans* mechanosensory neurons, with the bilateral pair of PLM neurons in the tail of the animal. The red rectangle indicates the approximate location of the sections highlighted in **b**, which show representative images of mitochondria fluorescently labelled with a GFP reporter (*Pmec*-*4::MLS::GFP*) in the PLM axons. Green puncta indicate mitochondria. *drp*-*1(tm1108)***ii** have more elongated mitochondria compared to wild type, **iii***eat*-*3(ad426)* are unchanged, and **iv***fzo*-*1(cjn020)* show smaller, rounder mitochondria. Images are representative sections of *n* = 12 PLMs from 12 worms analysed per genotype; scale bars 10 μm. **c** Mean size (µm^2^) of mitochondria in the PLM axon as determined using object segmentation (SQUASSH) at L4 stage. **d** Mean circularity of mitochondria in the PLM axon at L4 stage. **e** Mean size (µm^2^) of mitochondria in the PLM axon in 3DOAs. **f** Mean circularity of mitochondria in the PLM axon in 3DOAs. **g** Mitochondrial load: the total area (µm^2^) of mitochondria per PLM, normalised to PLM length. **h** Histogram and Gaussian distribution showing the variance of mitochondrial size in 3DOAs. All three genotypes show a significantly difference in variance (*F* test, *P* < 0.01) compared to WT. **i** Histogram and Gaussian distribution showing the variance of mitochondrial circularity in 3DOA. *F* test was performed on variance differences for both mitochondrial size and circularity. In all graphs; red = WT, yellow = *drp*-*1(tm1108)*, green = *eat*-*3(ad426)*, and blue = *fzo*-*1(cjn020)*. Data is represented as mean ± SEM. ^ns^*P *> 0.05, ****P < 0.0001 from one-way ANOVA with Dunnett’s post hoc tests for multiple comparisons; *n* ≥ 330 mitochondria for quantitative analysis, *n* ≥ 6 worms

To determine any tissue-specific differences and to assess how the absence of the fission and fusion proteins affects the nervous system, we next analysed mitochondrial morphology within the *C. elegans* mechanosensory neurons. We specifically focused on the bilateral pair of posterior lateral microtubule (PLM) neurons located in the tail of the animal (Fig. 5a), as these possess large axons embedded in the skin that are readily visualised using specific transgenes. We labelled mitochondria within the PLMs using a *Pmec*-*4::mitochondrial*-*GFP* transgene (Fig. 5b) and again quantified size and circularity using the SQUASSH segmentation ImageJ macro [26] (Supplementary Fig. 1C; Supplementary Table 1). Analysis of L4 stage animals showed that *drp*-*1* mutants have significantly increased mitochondrial size and significantly decreased mitochondrial circularity in the PLM axons (Fig. 5c, d). Mitochondria in the fusion mutants, *eat*-*3(ad426)* and *fzo*-*1(cjn020),* were similar in size and shape to wild type at this age (Fig. 5c, d). Analysis of older (3DOA) animals similarly revealed that mean mitochondria size was significantly increased, and mean circularity significantly decreased in *drp*-*1* mutants (Fig. 5e, f). In contrast, mitochondria in *fzo*-*1* mutants were significantly smaller and displayed significantly increased circularity compared to wild type (Fig. 5e, f). Surprisingly, *eat*-*3* mutants were not significantly different to wild type animals in the PLM neurons (Fig. 5e, f). To analyse any differences in variances we binned the values for both size and circularity in histograms and fitted a Gaussian distribution. At the 3DOA stage, the range of mitochondrial sizes and circularity was significantly wider for *drp*-*1* mutants, and narrower for *fzo*-*1* mutants compared to wild type (Fig. 5h, i). Animals mutant for *eat*-*3* had a wider (*F* test, *P* < 0.01) variance with respect to mitochondrial size compared to wild type (Fig. 5h), but showed no difference in mitochondrial circularity (Fig. 5i; *F* test, *P* = 0.21). In summary, and as expected, the loss of mitochondrial fission caused an increase in mitochondrial size and a decrease in circularity in the PLM axons, whereas disruption of outer membrane fusion resulted in smaller and more circular mitochondria. However, lack of inner membrane fusion did not cause significant changes to the morphology of mitochondria within these neurons.

Overall, our data reveal surprising tissue-specific differences in mitochondrial morphology in the absence of the fission and fusion machinery. Loss of fission induced more profound effects in neurons than in muscles, whereas loss of the fusion proteins displayed the opposite trend, with more prominent changes in mitochondrial morphology observed in muscles (compare Figs. 1 and 5).

### Mitochondrial dynamics proteins are crucial for maintaining neuronal function

**Fig. 6:**
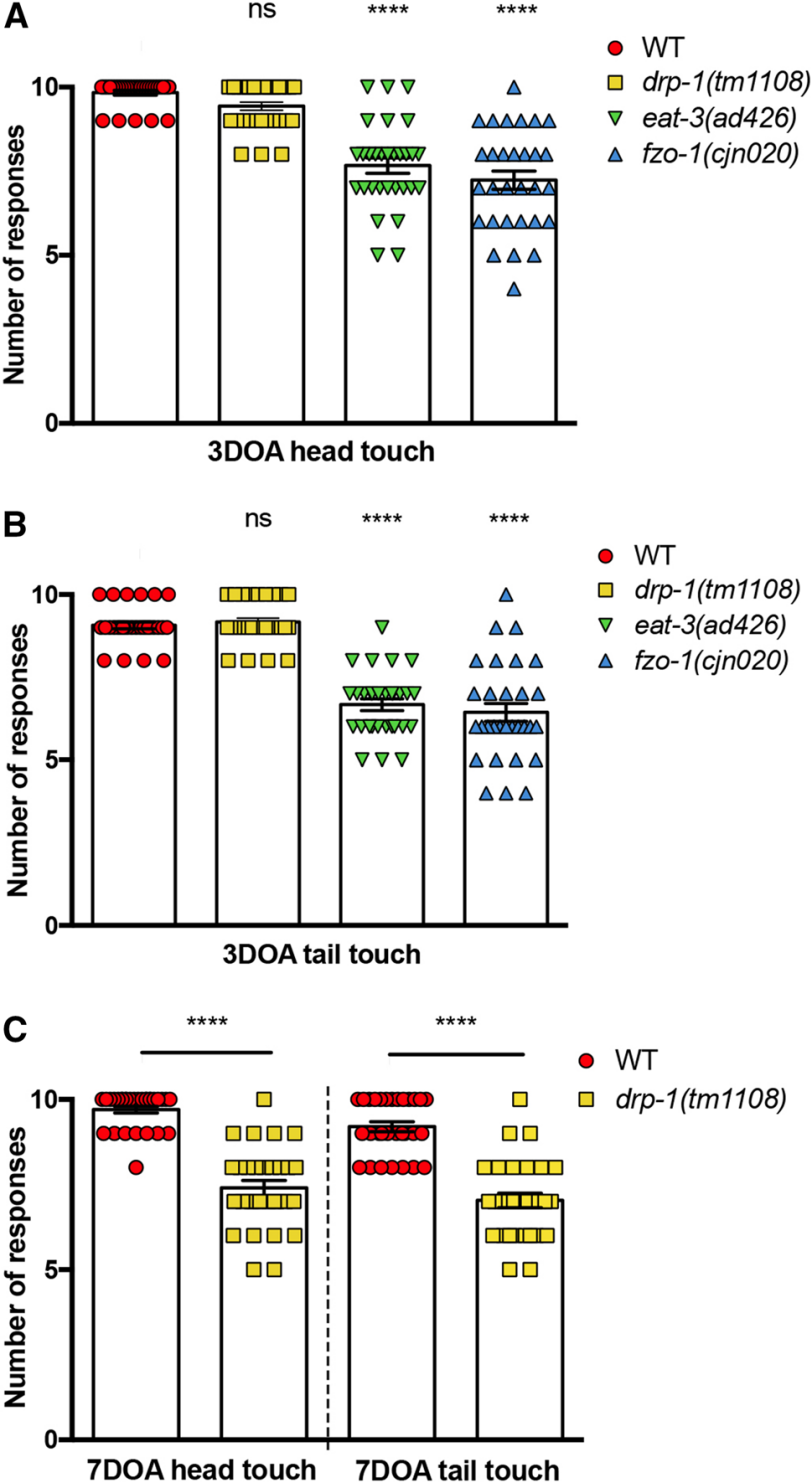
Mitochondrial dynamics proteins are crucial for maintaining neuronal function. **a** Quantification of anterior mechanosensory neuron function. Light touch assays performed by gently stroking worms across the head with an eyebrow hair and their response recorded. At 3DOA stage, *drp*-*1(tm1108)* mutants show no significant reduction in response to head touch. *eat*-*3(ad426)* and *fzo*-*1(cjn020)* both show significant reductions in response. Symbols represent individual animal responses from three replicate experiments; *n* ≥ 30 worms. **b** Quantification of posterior mechanosensory neuron function using the light touch assay. In 3DOAs, *drp*-*1(tm1108)* mutants show no significant reduction; *eat*-*3(ad426)* and *fzo*-*1(cjn020)* both show reductions in response; *n* ≥ 30 worms. **c***drp*-*1(tm1108)* shows a reduction in head and tail touch at 7DOA stage; *n* ≥ 30 worms. Bars represent mean, ± SEM; ^ns^*P *> 0.05, ****P < 0.0001 from one-way ANOVA with Dunnett’s post hoc tests for multiple comparisons

Upon establishing that disruptions in mitochondrial dynamics induce deficits in worm movement and muscle function, we next assessed whether neuronal function is also affected. We performed light touch assays [37, 38], which entail the gentle stimulation of the mechanosensory neurons in the head (ALMs and AVM) and the tail (PLMs) of animals with an eyebrow hair and recording backward or forward responses. At the 3DOA stage, both fusion mutants showed reduced responses to gentle mechanical stimulation: *eat*-*3* mutants presented a 22% reduction for head touch and 24% for tail touch; *fzo*-*1* mutants were 26% reduced for head touch and 33% reduced in tail touch (Fig. 6a, b). Although *drp*-*1* mutants showed no significant difference at 3DOA (Fig. 6a, b), we again observed defects at older ages in these animals, with reductions in mechanosensation observed in 7DOAs (Fig. 6c). These results demonstrate that the mitochondrial fission and fusion proteins are essential for neuronal function, with fusion required throughout life and fission only required at later stages of adulthood. Interestingly, these functional deficits do not directly correlate with changes in mitochondrial morphology: animals lacking either *eat*-*3* or *fzo*-*1* displayed no differences in mitochondrial morphology at the L4 stage, but displayed significant deficits in neuronal function at this age; *drp*-*1* mutants presented significant changes in mitochondrial morphology at both the L4 stage and in early adulthood, but neuronal function remained unaffected at these ages.

### Phenotypes induced by disruption of mitochondrial fusion can be rescued by the loss of fission

**Fig. 7:**
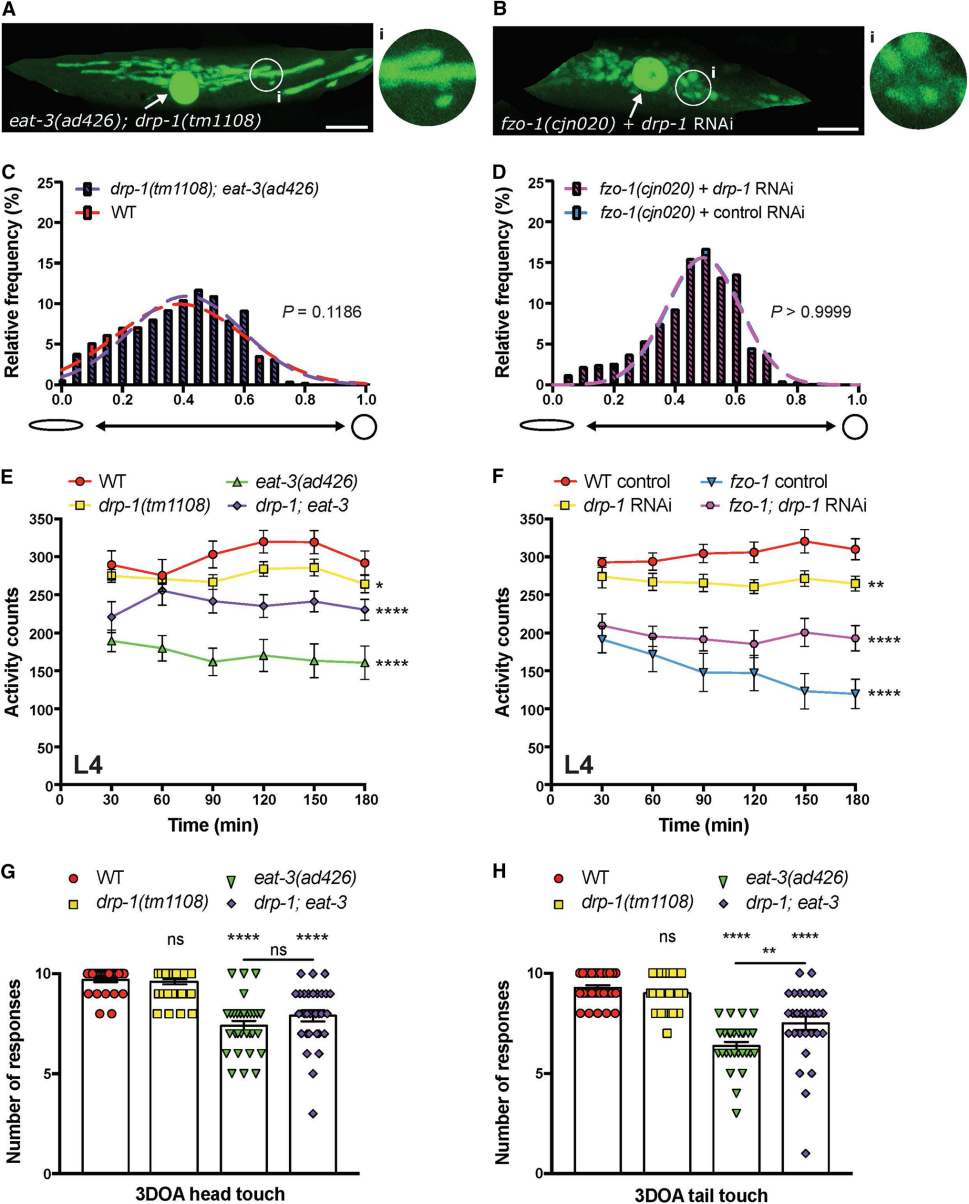
Simultaneous disruption of fusion and fission proteins rescues mitochondrial and behavioural phenotypes. Images of a body wall muscle with fluorescently labelled mitochondria (*Pmyo*-*3::MLS::GFP*) in **a** an *eat*-*3(ad426); drp*-*1(tm1108)* double mutant animal and **b** a *fzo*-*1(cjn020)* animal with RNAi knockdown of *drp*-*1*. Arrows designate nuclei, which are also labelled with GFP in this transgenic strain. Insets (**i**) are enlargements (× 3) of the indicated circular areas. Scale bars 20 μm. **c**, **d** Histogram and Gaussian distribution of mitochondria circularity scores for *eat*-*3(ad426); drp*-*1(tm1108)* double mutants (**c**) and *fzo*-*1(cjn020)* animals with RNAi knockdown of *drp*-*1* (**d**). All imaging was performed on L4 stage animals; *P* values calculated using *F* tests for variances; *n* ≥ 1832 mitochondria for quantitative analysis. **e**, **f** Quantified movement across populations of L4-stage animals using a WMicrotracker instrument. Data represents the mean ± SEM across ten individual wells; *n* ≥ 600 worms per genotype. *P < 0.05, **P < 0.01, ****P < 0.0001 compared to WT from one-way ANOVA with Dunnett’s post hoc tests. **g** Quantification of anterior mechanosensory neuron function using light touch assays. Symbols represent individual animal responses from three replicate experiments; *n* ≥ 30 worms. **h** Quantification of posterior mechanosensory neuron function using the light touch assay; *n* ≥ 30 worms. Bars represent mean, ± SEM; ^ns^*P *> 0.05, **P < 0.01, ****P < 0.0001 from one-way ANOVA with Dunnett’s post hoc tests for multiple comparisons

Previous research has demonstrated that mitochondrial fragmentation defects associated with loss of the fusion machinery are dependent upon mitochondrial fission [4, 22, 25, 39, 40]. Here, we have used our non-subjective methods to quantify the effects of simultaneous loss of both fission and fusion proteins on mitochondrial morphology, as well as on animal movement and neuronal function. To do so, we generated a strain carrying both the *drp*-*1(tm1108)* and *eat*-*3(ad426)* mutations. However, as we were not able to generate animals carrying mutations in both *drp*-*1(tm1108)* and *fzo*-*1(cjn020)* (likely a result of this combination being non-viable), we have used an RNAi approach. Remarkably, the morphology of mitochondria in the *drp*-*1*; *eat*-*3* double mutants was indistinguishable from wild type animals (Fig. 7a, c), demonstating that the deletion of DRP-1 can rescue the defect caused by the loss of EAT-3. However, knockdown of *drp*-*1* in *fzo*-*1(cjn020)* mutant animals could not do the same, with  no effect on mitochondrial morphology observed compared to *fzo*-*1(cjn020)* mutants (Fig. 7b, d; Supplementary Fig. 4C, D).

As shown in Fig. 7e, f, disruption of *drp*-*1* significantly rescued the movement defects caused by loss of either *eat*-*3* or *fzo*-*1*. Thus, despite the knockdown of *drp*-*1* having no effect on mitochondrial morphology in the *fzo*-*1(cjn020)* background, it could still improve animal movement. This further supports the lack of correlation between mitochondrial morphology and function. Furthermore, mechanosensory neuron function was also improved in *eat*-*3* mutants by the introduction of the *drp*-*1(tm1108)* mutation (Fig. 7g, h). These data support the notion that defects arising from loss of the fusion genes are partially dependent upon mitochondrial fission, and imply that by returning mitochondria to a neutral-like state without fusion and fission is favourable to one without appropriate fusion for animal behaviour.

### Disruption of mitochondrial dynamics shortens median, but not maximal lifespan

**Fig. 8:**
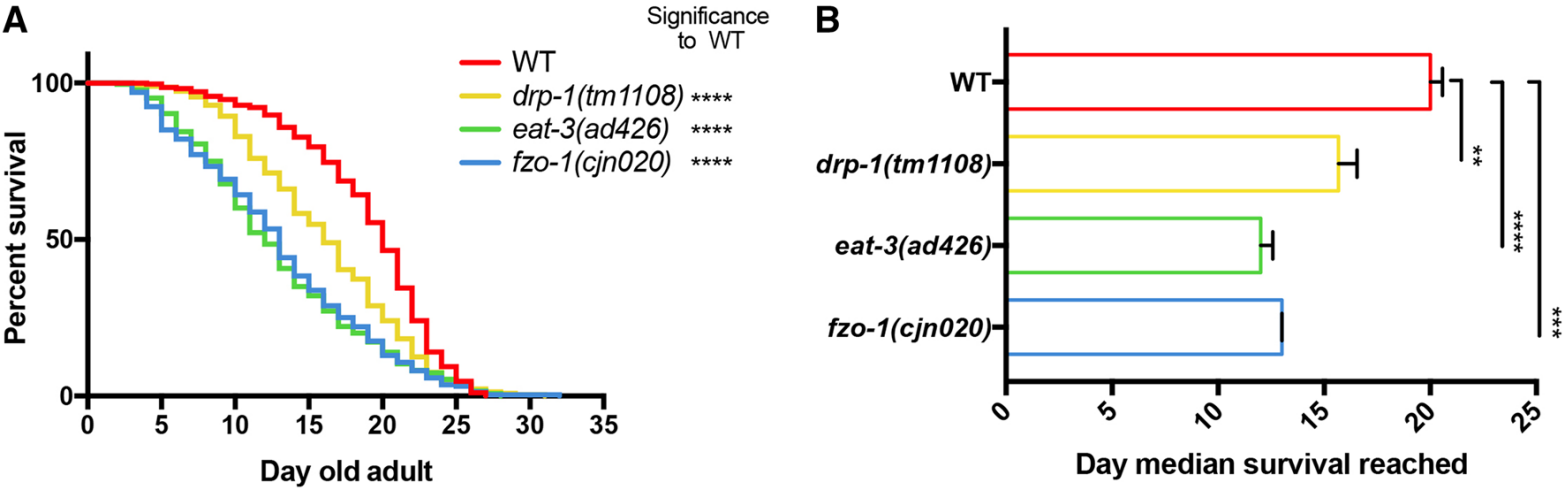
Reduced lifespan in the mitochondrial fusion/fission mutants. **a** Kaplan–Meier survival plot of mitochondrial fission/fusion mutants. All mutants (*drp*-*1(tm1108), eat*-*3(ad426)* and *fzo*-*1(cjn020)*) show reduced survival (*P *< 0.0001) compared to the wild type {strain QH3135 [*zdIs5(Pmec*-*4::GFP)*]}. **b** Median survival day of each mutant. *eat*-*3(ad426)* and *fzo*-*1(cjn020)* have a median survival of 12 and 13 days, respectively; *drp*-*1(tm1108)* has a median survival of 15.6 days; all of which are significantly less than the median survival of the wild type (20 days). Data are represented as the average of the median of each replicate, ± SEM. **P < 0.01, ***P < 0.001, ****P < 0.0001 from log-rank (Mantel–Cox) tests to compare the curves; three replicates with *n* ≥ 207 worms per genotype (individual replicates are shown in Supplementary Fig. 4)

Given the significant reductions observed in muscle and neuronal function, we assessed how disruptions in mitochondrial dynamics affect animal lifespan. We performed longevity assays [41] in triplicate in wild type, *drp*-*1(tm1108)*, *eat*-*3(ad426)* and *fzo*-*1(cjn020)* strains. Interestingly, all three mutant backgrounds displayed significantly reduced median lifespan when compared to wild type animals, but maximal lifespan remained unchanged (Fig. 8, Supplementary Fig. 5). The fusion mutants (*eat*-*3* and *fzo*-*1*) presented near identical survival curves (Fig. 8a) and median survival of 12 and 13 days, respectively (Fig. 8b). Mutation of *drp*-*1* caused an intermediate defect, with a median survival of 15.6 days, significantly reduced to that of the wild type (20 days) (Fig. 8b). The similarity in maximal survival, but reduced median survival for all strains, indicates that disrupting mitochondrial dynamics has detrimental effects on the health of an organism, leading to significant variance in lifespan.

## Discussion

Our results show that loss of the proteins controlling mitochondrial fission and fusion induces tissue-specific morphological changes to mitochondria and significantly diminishes the normal health and behaviour of *C. elegans*. These findings highlight the important role that fission/fusion processes have within the context of a whole organism and provide new insights into the consequences of imbalances in mitochondrial dynamics that are associated with various human diseases.

Optimisation of the sophisticated segmentation protocol developed by Rizk et al. [26] allowed us to accurately assess size and shape on an individual mitochondrion or connected network basis, providing non-subjective methodology for quantification of mitochondrial morphology. This approach confirmed that mitochondrial dynamics are altered in the absence of *drp*-*1*, *eat*-*3* and *fzo*-*1,* and uncovered unexpected tissue-specific effects. In *C. elegans* muscle cells, absence of the proteins required for either inner or outer membrane fusion induced similar fragmented phenotypes, with smaller and more circular mitochondria phenotypes consistent with previous studies [21, 42]. Reduced mitochondrial networks and size are associated with decreased function [5], suggesting that the bioenergetic capacity of muscles cells in the absence of fusion is sub-optimal. Somewhat surprisingly, when we blocked mitochondrial fission by knocking out *drp*-*1*, we did not see an increase in elongated mitochondrial networks in muscles. These findings are, however, consistent with recent studies in *C. elegans* that have also not observed hyper-connected mitochondria when using genetic knockouts [5, 27]. Ackema et al. demonstrated that the absence of DRP-1 in fact causes the formation of blebs in the mitochondrial network, which may be a consequence of fission of the inner mitochondrial membrane still occurring in the absence of DRP-1 [24, 27, 28]. In mammalian cells, and in concert with DRP1, the dynamin-2 protein was recently shown to drive the final constriction event required for mitochondrial division [43]. Knockdown of the sole dynamin family orthologue in *C. elegans* (DYN-1) has been reported to disrupt mitochondrial morphology [44]. Fission can also occur independently of DRP1 in certain conditions, with autophagosomes shown to pinch off mitochondrial segments to promote mitophagy [45]. Thus, the blebbing phenotype observed in muscle cells lacking DRP-1 may be a consequence of disrupting the coordinated activities of DRP-1 and DYN-1, or an effect of DRP-1-independent mitochondrial fission in this tissue.

Our ultrastructural analysis of mitochondria in the *C. elegans* body wall muscles revealed a role for FZO-1/mitofusin 2 in the correct organisation of cristae. The majority of mitochondria displayed severe reductions in the density of cristae and aberrations in their appearance, with long, narrow structures frequently seen. This was unanticipated, as it is the inner mitochondrial membrane fusion protein EAT-3/OPA1 (not FZO-1/mitofusin 2) that has a well-established role in cristae organisation and remodelling [30, 46]. We are not aware of any other studies directly linking mitofusin 2 with cristae structure. However, coordination between OPA1 and mitofusin 2 in cristae remodelling has been previously demonstrated [47]. This study identified interactions between the molecular components controlling cristae organisation (including OPA1) and mitofusin 2-dependent mitochondria–endoplasmic reticulum (ER) tethering as important determinants of cristae remodelling. Whether the dramatic changes in cristae structure we observed in FZO-1/mitofusin 2 null mitochondria are therefore related to interaction with EAT-3/OPA1 and/or its role as a mitochondrial–ER tether remain to be determined. Nevertheless, our data establishes the importance of FZO-1/mitofusin 2 in organisation of cristae structure in *C. elegans*.

The changes in mitochondrial morphology in the muscles cells were associated with significant reductions in motor activity and muscle strength (WMicrotracker, thrash, body bending and burrowing assays). Similar to sarcopenia observed in humans, *C. elegans* display a progressive loss of muscle mass and function with age [48]. Mitochondrial dysfunction has been hypothesised to be a major driver of this phenotype [49], and previous studies have linked changes in mitochondrial morphology to sarcopenia [50–53]. However, whether these are a cause or a consequence of age-dependent muscle decline remains controversial [49, 50]. Our results conclusively demonstrate that disruption of either mitochondrial fusion or fission causes severe defects in muscle function in *C. elegans*, highlighting the importance of a correct fusion/fission balance for muscle health.

In contrast to our findings in muscle cells, where we observed significant changes in mitochondrial size and circularity without EAT-3 or FZO-1 but not with loss of DRP-1, our quantification of mitochondrial morphology in the nervous system revealed the opposite trend. This was especially evident at the L4 stage, where DRP-1 mutants displayed a twofold increase in the mean size of mitochondria and a significant reduction in mean circularity, whereas animals lacking either fusion gene displayed no change compared to the wild type. The physical constraints of the PLM axon may in part explain these tissue-specific differences, as larger fused mitochondria are confined into elongated shapes within the axon, as opposed to the more three-dimensional matrix present within the muscle cells. Alternatively, differences in energy demands between these tissues may result in altered requirements for mitochondrial dynamics that may therefore contribute to the tissue-specific variations. While intuitively increased mitochondrial networks may be considered an advantage to neuronal function, we still observed a decrease in mechanosensation in older DRP-1 null animals, again suggesting that disrupting the fusion/fission balance in either direction can have detrimental effects. Indeed, the importance of balanced mitochondrial fission and fusion was recently underscored by Chen et al. who showed that loss of fusion can rescue tissue and lifespan defects associated with disruption of fission [54]. Our data provide further support to this notion, revealing that disrupting fission can inhibit the deficits in mitochondrial morphology, animal movement and neuronal function associated with loss of the fusion genes. Thus, an appropriate balance in mitochondrial dynamics may be more important than the correct regulation of either fission or fusion.

Although morphological alterations varied between tissues, overall changes in animal behaviour in the absence of fission/fusion were more consistent. This indicates that mitochondrial morphology and function are not always tightly correlated. Despite extensive evidence in mammalian cells showing that disruption of the fusion and fission genes directly affects mitochondrial function [9, 55, 56], recent findings in *Drosophila melanogaster* have demonstrated that morphology and function can be dissociated [57]. While morphology was found to be crucial for the proper axonal distribution of mitochondria, neuron health was solely dependent on the bioenergetic capacity of mitochondria and not their shape [57]. Our data support these findings by demonstrating that changes in mitochondrial morphology do not consistently correlate with impaired behaviour in a whole animal model.

Our longevity assays revealed that loss of either DRP-1, EAT-3, or FZO-1 significantly reduces median lifespan, but does not affect maximal lifespan. This suggests that the mitochondrial dynamics proteins are all required for limiting variance in population health. Through the analysis of a massive number of animals (> 100,000 individuals), Stroustrup et al. demonstrated that the dynamics of ageing in *C. elegans* is largely invariant across diverse interventions that extend or shorten lifespan [58]. Changes in diet, environmental conditions and genetic lesions all acted to stretch or shrink the survival curves along the time axis, implying that ageing is governed by temporal scaling. However, despite this invariance across the majority of conditions tested, this study identified two genes that altered the shape of the survival curve when mutated. Intriguingly, one of these altered mitochondrial function (mutation of *nuo*-*6*, encoding a component of mitochondrial respiratory chain complex I). Mirroring our findings in animals lacking DRP-1, EAT-3, or FZO-1, mutation of *nuo*-*6* reduced median survival, but left maximal survival largely unaffected [58]. Moreover, mitochondrial fusion has been shown to be critical for the lifespan extension associated with a diverse range of longevity pathways [59]. Thus, our data strongly support these previous studies, and together suggest that disruptions in mitochondrial health and dynamics have significant effects on lifespan by increasing the variance of survival.

*Caenorhabditis elegans* are kept largely in isogenic populations due to their hermaphroditism, invariant developmental cell lineages [60, 61], and standardised growth conditions. However, these genetically identical individuals routinely display phenotypic variation, a phenomenon shared by most if not all organisms [62]. In *C. elegans*, this variability has been attributed to stochastic fluctuations in both gene expression [63] and genetic compensatory responses to mutations [64], consequent variation in stress signalling [65], and more recently to maternal age [66]. Perez et al. demonstrated that progeny from young mothers exhibit impairments across a range of phenotypic traits compared to progeny from old mothers. Age-dependent changes in the provision of yolk proteins to embryos was identified as the major cause of these differences and was therefore proposed as a primary driver of phenotypic variation [66]. Disruption of either DRP-1, EAT-3, or FZO-1 is associated with reductions in brood size and growth rates in *C. elegans* [4, 67–70]. Thus, it is plausible that these defects impact on the maternal age of the animals, and that this may drive the variance in survival across populations defective in mitochondrial dynamics.

Consistently across our different behavioural assays, we observed significantly reduced responses for animals lacking DRP-1 during development, no defect in early adulthood, and significant reductions again in older animals. Loss of EAT-3 or FZO-1 induced more consistent and progressive defects across development and adulthood. These trends show that the importance of mitochondrial fission fluctuates with age and imply that fusion has a homeostatic role in mitochondrial health, whereas fission is needed predominantly later in life, possibly to combat ageing-associated stressors. Given that a major role of fission is to facilitate the clearance of defective mitochondria, this may be a reflection of changes in the requirements of mitophagy, which are known to vary with age [71]. The increased severity of defects in the absence of the fusion proteins also implies that in terms of tissue health, mitochondrial fusion is more important than mitochondrial fission. Thus, the generation of connected mitochondrial networks is more critical than breaking these apart. This is further supported by the close association of mutations in *MFN2* and *OPA1* with Charcot–Marie–Tooth disease and optic atrophy, respectively [7, 12–14], while disease as a direct consequence of *DRP1* mutation occurs far less frequently [16]. Whether this relates to the major function of fusion in mitochondrial functional complementation [1] remains to be determined.

In conclusion, we reveal new insights into the importance of the mitochondrial dynamics proteins in controlling organismal health. We uncover previously underappreciated tissue-specific roles for mitochondrial dynamics, a role for FZO-1/mitofusin 2 in cristae structure, different requirements for the key proteins across development and adulthood, and novel roles for mitochondrial fission and fusion in the temporal scaling associated with ageing.

## Materials and methods

### Generation and maintenance of *C. elegans* strains

Maintenance, crosses, and other genetic manipulations were all performed via standard procedures [72]. Hermaphrodites were used for all experiments and were grown at 20 °C on nematode growth medium (NGM) plates seeded with OP50 *Escherichia coli*. The *drp*-*1(tm1108)*, *drp*-*1(or1393), dys*-*1(eg33), eat*-*3(ad426)*, *eat*-*3(tm1107)*, *fzo*-*1(cjn020)* and *fzo*-*1(tm1133)* mutations were used together with the following transgenes: *jsIs609(Pmec*-*4::MLS::GFP)* [73], *ccIs4251(Pmyo*-*3::GFP::LacZ::NLS, Pmyo*-*3::mitochondrial-GFP *+* dpy*-*20(*+*))* [74], *uIs115(Pmec*-*17::tagRFP)* [75], and *zdIs5(Pmec*-*4::GFP)*. A full list of strains is shown in Supplementary Table 2.

The *fzo*-*1(tm1133)* strain contains an uncharacterised background mutation that induces morphological defects in the nervous system that are not related to the mutation of *fzo*-*1* (unpublished observations). As such, we generated a new deletion allele [*fzo*-*1(cjn020)*] with CRISPR/Cas9 using double-stranded breaks and non-homologous end joining [76]. This strain carries a deletion of 2629 bp covering nucleotides 25-2654 of the *fzo*-*1* gene.

### Fluorescence imaging

For confocal imaging of mitochondria, *C. elegans* carrying the *ccIs4251* transgene were used for analysis of body wall muscles and the *jsIs609* transgene were used for analysis of the PLM axons. Worms were immobilised with 0.05% solution of tetramisole hydrochloride and mounted on 4% agarose pads on glass slides. Imaging was carried out using a Leica SP8 inverted HyD confocal microscope (63 × objective with 1.4 NA oil immersion for muscles; 40 × objective with 1.4 NA water immersion for PLMs) and under non-saturating exposure conditions and running LAS AF software (Leica MicroSystems. Mannheim, Germany). Images of mitochondrial morphology in body wall muscles were taken at L4 stage from the upper or lower part of the worm, excluding the regions adjacent to the oesophagus and vulva. Tile scans were taken to capture the entire PLM axon in L4 and 3DOA animals.

### Quantitative measurement of mitochondrial size and shape

We processed Leica Image Files (.lif) into .tiff images and performed maximum intensity projections on *Z*-stacks using Fiji image software [77]. We then performed split-Bregman segmentation [78] using the Mosaic Suite SQUASSH (segmentation and quantification of subcellular shapes) ImageJ plugin (http://mosaic.mpi-cbg.de/), as previously described [26]. In short, this involves removing background noise, detecting and separating objects (mitochondria) within an image, followed by estimation of the local background and object intensities, which aid in the computation of optimal segmentation of each object. The parameters used for the segmentation of mitochondria within both the body wall muscle cells and PLM axons are displayed in Supplementary Table 1. An example for each segmentation is demonstrated in Supplementary Fig. 1A, C. For each set of parameters, manual observation of segmented objects was used to determine if parameters provided an accurate segmentation of mitochondria. Following accurate segmentation, object characteristics for size, perimeter, and length were measured using the plugin, and then used to calculate values for shape. We used a two-dimensional measure of sphericity, “circularity”, to estimate mitochondrial shape. Circularity is a function of area and perimeter [Circularity = (4 × *π*) × (Area/Perimeter^2^)] which fits each object to a perfect circle and measures its deviation, where 1 = a perfect circle and 0 = a straight line. For mitochondria in the body wall muscle, the data was subset to exclude objects with a size < 5 pixels to remove potential noise not picked up by the plugin. Histograms and Gaussian distributions were calculated using GraphPad Prism software (Version 7.0a for Mac), using bins of 0.2 µm for mitochondrial size and 0.05 for mitochondrial circularity. Mitochondrial load was calculated as the total area of mitochondria per PLM, normalised to wild type based on the average PLM length per genotype.

### Transmission electron microscopy

L4 stage animals were placed into 3 mm freezer hats (200 µm depth) in a cryoprotectant consisting of 20% BSA and OP50 *E. coli*. They were subsequently frozen using a Leica EM PACT 2 high-pressure freezer and stored in liquid nitrogen. Freeze-substitution was performed in a Leica EM AFS2 unit in flat-bottomed BEEM capsules using methods modified from those previously published [79]. Samples were incubated at − 90 °C for 48 h in 0.1% tannic acid and 0.5% glutaraldehyde. They were then rinsed with pre-cooled acetone four times over 4 h, before being placed into 2% OsO_4_ in acetone for 4 h at − 90 °C. Samples were brought to − 20 °C over a period of 14 h and incubated for a further 14 h at this temperature. Samples were brought to 4 °C over a period of 5 h after which they were rinsed four times with fresh acetone. Samples were then brought to room temperature over a period of 2 h and removed from the AFS unit. Substituted discs of worms in cryoprotectant were removed from freezer hats using fine titanium needles and transferred to glass vials in acetone. The acetone was replaced with 100% propylene oxide in preparation for resin embedding and incubated overnight at room temperature. The samples were infiltrated in a graded series of Epon/Araldite in propylene oxide consisting of 1:1, 2:1 and 3:1 resin to propylene oxide for 24 h for each concentration. The samples were then incubated in 100% resin two times for 24 h each. Following infiltration sample discs were placed into flat embedding moulds with fresh resin and polymerised in an oven at 60 °C for 48 h.

Embedded sample discs were sectioned with a Leica Ultracut UCT ultramicrotome using a Diatome diamond knife, and 90 nm thick sections were collected onto formvar-coated copper slot grids. The sections on grids were sequentially stained with 1% aqueous uranyl acetate and lead citrate. Cross sections of nematodes were located and imaged with a JEOL 1400 + transmission electron microscope at 80 kV. Digital images of mitochondria located immediately below muscle sarcomeres were taken at a resolution of 2 K × 2 K in TIFF and JPEG format. Images were obtained from one animal per genotype.

### Behavioural assays

#### WMicrotracker

Large populations of age-synchronised worms were prepared according to the protocol devised by Solis and Petrascheck [80]. Briefly, gravid hermaphrodites were bleached to release eggs, which were allowed to hatch overnight before plating onto OP50-seeded 60 mm NGM plates and incubation at 20 °C until the day of experiment. Synchronised L4 or 3DOA worms were initially washed off the plates with M9 buffer into micro-centrifuge tubes, and subsequent washing of the worms in the tubes was performed at least twice to remove residual OP50 and offspring. For analysis at L4 stage, 100 μL of 60–70 L4 worms was then plated into five wells per genotype of a 96-well plate across three replicate experiments. For analysis of adults, 30–50 3DOA worms were plated into six wells of a 96-well plate per genotype, across two replicate experiments. The plates containing the worms were placed in a WMicrotracker instrument (Phylum tech [31]) and run for 3 h.

#### Thrashing assay

Age-synchronised worms were transferred to a fresh unseeded plate at room temperature to remove leftover OP50; then individual worms were transferred to a 10 μl droplet of M9 buffer [72]. Worms were allowed to acclimatise for 15 s, and the number of completed thrashes per minute was counted using a hand counter. This was performed on ten individual worms per replicate, 3 × replicates in total. All assays were performed at room temperature.

#### Body bends

Individual age-synchronised worms were gently placed on unseeded 60 mm NGM plates and left to acclimatise for 3–5 min at room temperature. The number of complete body bends, defined as the maximum bend of the part of worm just behind the pharynx from one end to the opposite direction in a forward sinusoidal pattern, was manually counted for 3 min [81]. The reverse bend in the same direction was not included in the count. The number of body bends per minute was then averaged.

#### Exploration assay

The assay was performed as previously described [34, 35]. Individual age-synchronised worms were picked on 60 mm NGM plates uniformly seeded with a thin layer of OP50 *E. coli*. The worms were then removed after 16 h incubation at room temperature and the plates were superimposed on a grid with 3.5 mm squares. The number of squares entered by the worm tracks, with a maximum of 86 squares, was manually counted.

#### Burrowing assay

Burrowing assays were conducted in 5 mL serological pipettes filled with chemotaxis agar at 1.5% as described previously [36]. Two holes were drilled on either end of the pipette, one for the worms and the other for an attractant (diacetyl, 2 μL of 1:100 dilution) to allow directed burrowing. Pipettes were stored at 4 °C overnight to promote gradient formation. 24 h later, ~ 50 worms were injected into one end of the pipette and the worms were allowed to burrow towards the attractant for 4 h. Afterwards, the position of each worm was viewed under a dissecting microscope and marked on the surface of the pipette. The number of worms present in each cm was counted and the percentage of overall worms to have travelled per cm was calculated cumulatively. The worms that burrowed towards the opposite side without the attractant or did not cross 0.5 cm from the injection site were excluded from quantifications.

#### Chemotaxis assay

Chemotaxis assays were performed in 10 cm Petri dishes containing 10 mL of chemotaxis agar (2% agar, 5 mM KPO_4_, 1 mM CaCl_2_, and 1 mM MgSO_4_). The plates were allowed to dry at room temperature for 24 h before the assay. Age-synchronised 1DOAs were washed thrice in M9 buffer and placed at the centre of the plate at equal distance from the attractant (diacetyl 1:1000) and control (100% ethanol) spots. 1 μL of 1 M sodium azide was applied to both the attractant and control spots and allowed to dry for 5 min. Animals were allowed to move over the assay plate for 1 h and then placed at 4 °C overnight. The distribution of the animals over the assay plate was counted the next day and animals that did not leave the inner circle were excluded from the analysis. Chemotaxis index (CI) was calculated using the formula: CI = (number of animals in the attractant area − number of animals in the control area)/(total number of scored animals). Chemotaxis assays were performed in duplicate for each genotype across three independent trials.

#### Light touch assay

To measure the function of the mechanosensory neurons ALM/AVM and PLM, we performed 20-trial touch assays using an eyebrow hair as previously described [37, 38]. Animals were touched a total of 20 times (alternating head and tail touches—10 touches each) and full, partial, and no responses recorded. At least 30 animals were examined and mean percentage scores obtained.

### Lifespan

Lifespan experiments were performed on *drp*-*1(tm1108)*, *eat*-*3(ad426)*, *fzo*-*1(cjn020)* and wild type {strain QH3135 [*zdIs5(Pmec*-*4::GFP)*]} as previously described [41]. ~ 10 L4 stage animals were placed on eight to ten plates per genotype, and assessed for mortality each day through observation of movement and pharyngeal pumping, and if necessary, light prodding. We performed our experiments in triplicate. Kaplan–Meier survival plots were generated using GraphPad Prism, and survival curves were compared using a log-rank (Mantel–Cox) test.

### RNAi

RNA interference of *drp*-*1* was performed using a feeding method [82]. Animals were grown on IPTG-containing NGM plates seeded with *E. coli* [*HT115(DE3)*] expressing dsRNA of *drp*-*1* or the vector control (L4440). Animals were grown on these plates for multiple generations prior to analysis.

### Statistical analysis

Statistical analysis was performed using GraphPad Prism 7. ANOVA was used for comparing groups with more than two samples followed by Dunnett’s multiple comparison post hoc test. *F* test was used to compare variances. Multiple *t* tests (one per row) using the Holm–Sidak method were used to compare muscle strength. A log-rank (Mantel–Cox) test was used to compare survival curves of the Kaplan–Meier survival plots.

## Electronic supplementary material

Below is the link to the electronic supplementary material.

Supplementary Fig. 1. SQUASSH image segmentation of mitochondria within body wall muscles and PLM axons. (**A**) Representative images of body wall muscle cells of a wild type worm before (left) and after (right) image segmentation using the SQUASSH ImageJ plugin. The white objects in the before image are mitochondria and mitochondrial networks. The right image shows outlines of mitochondria the plugin has found and successfully segmented from the image. Strongly fluorescent muscle cell nuclei removed to aid analysis. Scale bars = 20 μm. (**B**) Representative images of body wall muscle cells of *drp*-*1(tm1108), eat*-*3(ad426)* and *fzo*-*1(cjn020)* mutants. Scale bars = 20 μm. (**C**) Inset of PLM axon (3x zoom) of a wild type worm before (top) and after (bottom) image segmentation. The white objects in the before (top) image are mitochondria. Faint fluorescence in the background is the PLM axon. The bottom image shows outlines of mitochondria the plugin has found and successfully segmented from the image. Scale bars = 3 μm (JPEG 1343 kb)

Supplementary Fig. 2. Electron microscopy analysis of mitochondria. Each series of images show mitochondria captured with electron microscopy transverse sections from the body wall muscles of L4 stage animals. Images are shown for (**A**) wild type, (**B**) *drp*-*1(tm1108)*, (**C**) *eat*-*3(ad426),* and (**D**) *fzo*-*1(cjn020)* animals. Arrows point to inner membrane septae; asterisks to electron-dense inclusions; arrowheads to inclusion bodies; scale bars = 200 nm (JPEG 5098 kb)

Supplementary Fig. 3. Analysis of additional alleles of mitochondrial dynamics proteins, exploration behaviour, and muscle strength. (**A**) Quantification of the number of thrashes per minute in liquid. At 3DOA stage both the fusion mutants, *fzo*-*1(cjn020)* and *eat*-*3(ad426)*, and the fission mutant *drp*-*1(tm1108)* show no significant difference compared to the relevant secondary allele, *fzo*-*1(tm1133), eat*-*3(tm1107)* and *drp*-*1(or1393)*. (**B**) Number of thrashes per minute in liquid at 7DOA stage quantified. Both the fusion mutants, *fzo*-*1(cjn020)* and *eat*-*3(ad426)* and fission mutant *drp*-*1(tm1108),* show no significant difference compared to the relevant secondary allele, *fzo*-*1(tm1133), eat*-*3(tm1107)* and *drp*-*1(or1393)*. (**C**) Number of squares entered by each worm after 16 h as a representation of exploration. Symbols show individual animals from three replicate experiments; n ≥ 30 3DOA worms. Data is represented as the mean, ± SEM. (**D**) Chemotaxis indexes calculated for WT and mutant animals. Chemotaxis indexes were calculated for 1DOA animals exposed to attractant (diacetyl) and control (ethanol) for 1 h. (**E**) Muscle strength compared to a defective control. *fzo*-*1(cjn020)* shows a comparable reduction in muscle strength compared to known muscle defective mutant *dys*-*1*. Data is represented as mean cumulative distance covered ± SEM of three replicate experiments. n ≥ 36 worms. ns = *P *> 0.05, * = *P* < 0.05, ** = *P* < 0.01, *** = *P* < 0.001, **** = *P* < 0.0001 compared to WT unless indicated otherwise from one-way ANOVA with Dunnett’s post hoc tests for multiple comparisons (JPEG 1134 kb)

Supplementary Fig. 4. Mitochondrial number and PLM length in the absence of mitochondrial fusion/fission. (**A**) Number of mitochondria counted per PLM. *eat*-*3* shows a significant reduction in mitochondrial number. n = 12 PLM, 1 per worm. (**B**) PLM length between fission/fusion mutants. Fusion mutants, *fzo*-*1(cjn020)* and *eat*-*3(ad426)* both show a reduced PLM length. Symbols show individual PLMs, n = 12 PLM, 1 per worm. Data is represented as the mean, ± SEM. (**C**) Mean size (µm^2^) of mitochondria in the body wall muscles as determined using object segmentation (SQUASSH). (**D**) Mean circularity of mitochondria in the body wall muscles, calculated by fitting each object to a perfect circle and measuring deviation using the following formula (4 x π) x (Area/Perimeter^2^). A value of 1 represents a perfect circle, and 0 a straight line. ns = *P* > 0.05, ** = *P* < 0.01, *** = *P* < 0.001, **** = *P* < 0.0001 from one-way ANOVA with Dunnett’s post hoc tests for multiple comparisons (JPEG 2314 kb)

Supplementary Fig. 5. Reduced lifespan in the mitochondrial fusion/fission mutants, single replicate experiments. (**A-C**) The individual replicate Kaplan–Meier Survival Plots for the mitochondrial fission/fusion mutants. All mutants - *drp*-*1(tm1108), eat*-*3(ad426)* and *fzo*-*1(cjn020)* - show significantly reduced mean survival in all three replicates, with maximal survival unchanged. Data is represented as a survival curve of each replicate, ± SEM. * = *P* < 0.05, ** = *P* < 0.01, *** = *P* < 0.001, **** = *P* < 0.0001 from log-rank (Mantel-Cox) tests to compare the curves; n ≥ 60 worms per genotype for each replicate (JPEG 516 kb)

Supplementary material 6 (DOCX 41 kb)

Supplementary material 7 (DOCX 67 kb)
