# Supplementary figures and images for: Disruption of mitochondrial dynamics affects behaviour and lifespan in Caenorhabditis elegans
Source: Cell Mol Life Sci. 2019 Mar 6;76(10):1967–85. doi: 10.1007/s00018-019-03024-5 (PMC6478650; doi:10.1007/s00018-019-03024-5)

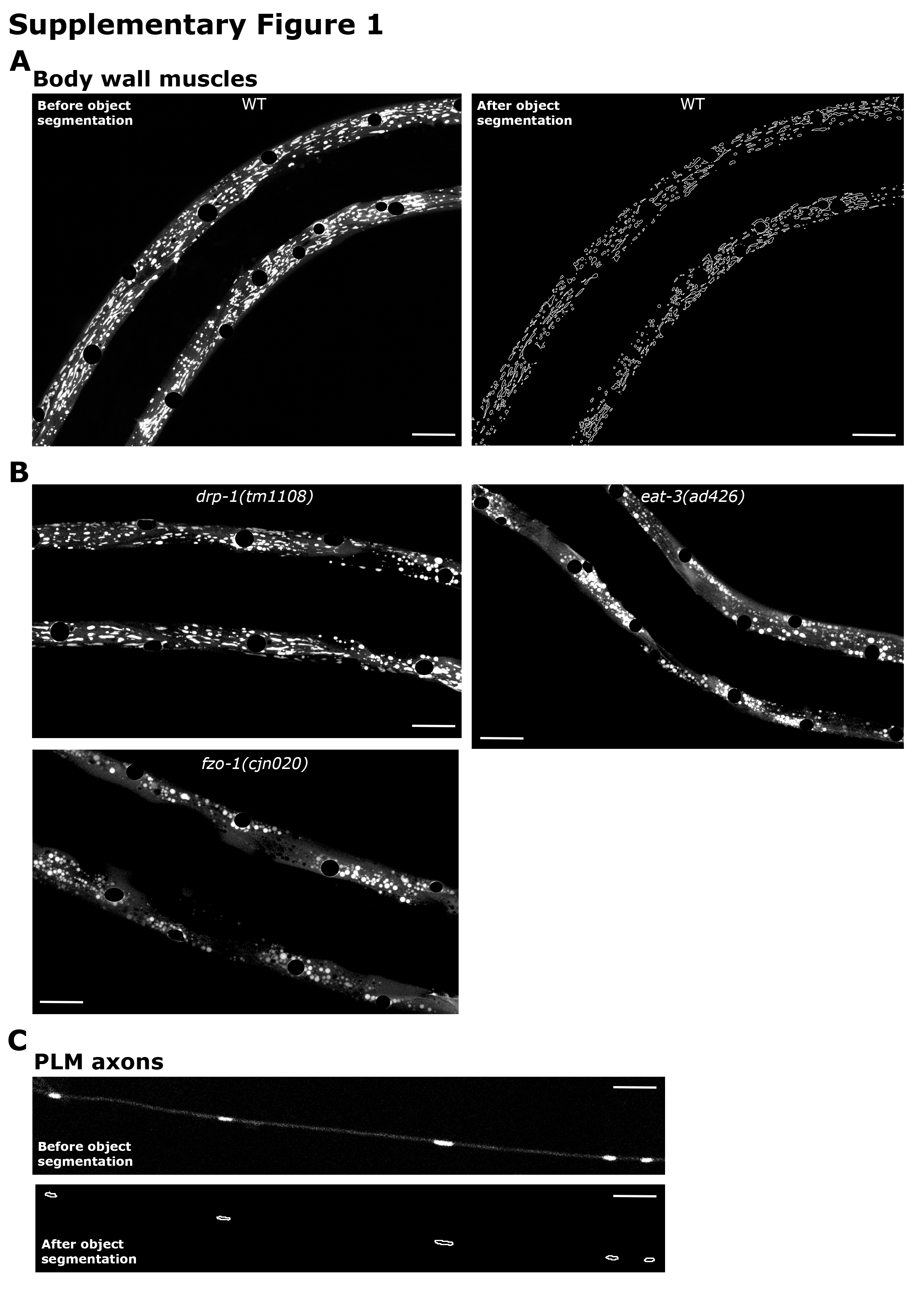

Supplement: Supplementary file 1 — Supplementary Fig. 1. SQUASSH image segmentation of mitochondria within body wall muscles and PLM axons. (A) Representative images of body wall muscle cells of a wild type worm before (left) and after (right) image segmentation using the SQUASSH ImageJ plugin. The white objects in the before image are mitochondria and mitochondrial networks. The right image shows outlines of mitochondria the plugin has found and successfully segmented from the image. Strongly fluorescent muscle cell nuclei removed to aid analysis. Scale bars = 20 μm. (B) Representative images of body wall muscle cells of drp-1(tm1108), eat-3(ad426) and fzo-1(cjn020) mutants. Scale bars = 20 μm. (C) Inset of PLM axon (3x zoom) of a wild type worm before (top) and after (bottom) image segmentation. The white objects in the before (top) image are mitochondria. Faint fluorescence in the background is the PLM axon. The bottom image shows outlines of mitochondria the plugin has found and successfully segmented from the image. Scale bars = 3 μm (JPEG 1343 kb) [file 18_2019_3024_MOESM1_ESM.jpg]

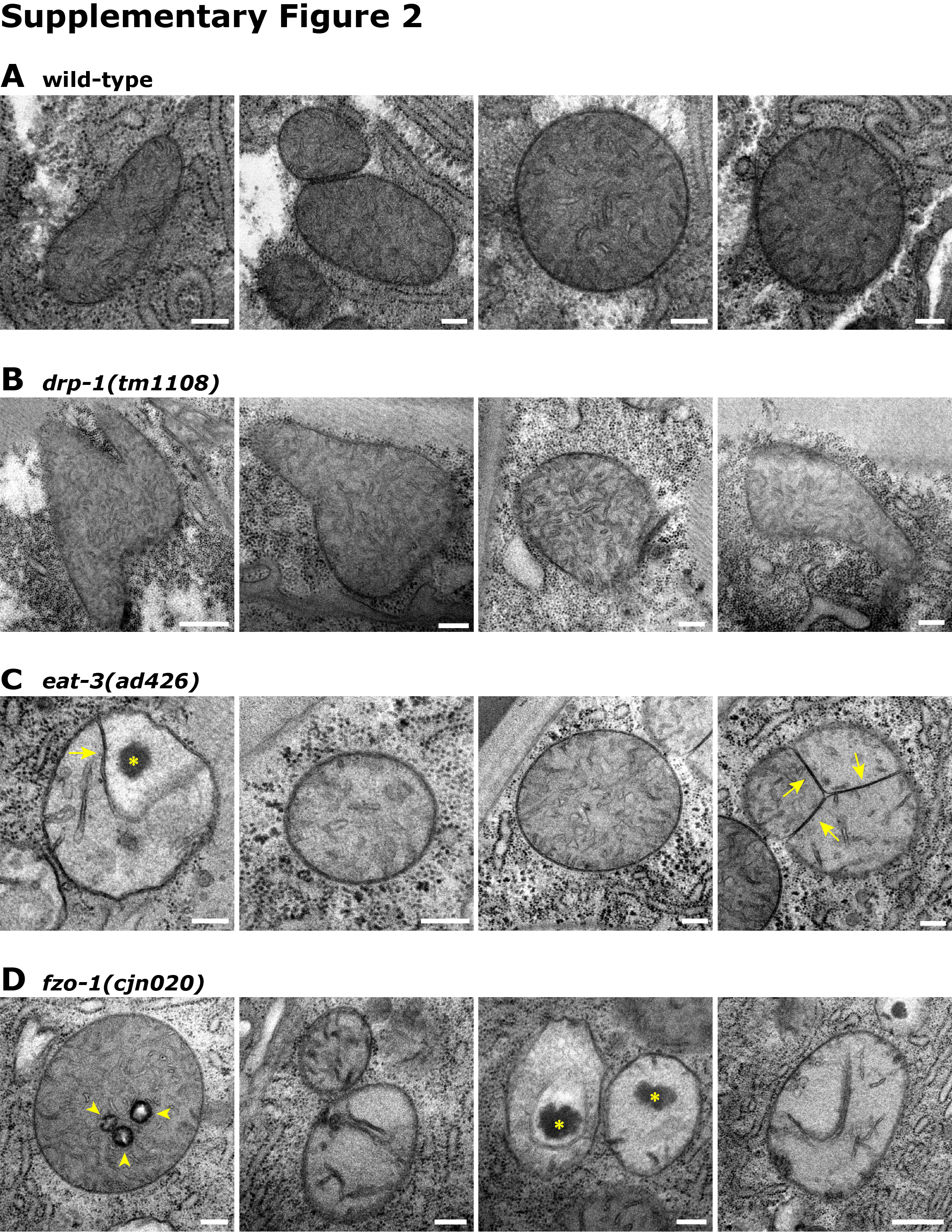

Supplement: Supplementary file 2 — Supplementary Fig. 2. Electron microscopy analysis of mitochondria. Each series of images show mitochondria captured with electron microscopy transverse sections from the body wall muscles of L4 stage animals. Images are shown for (A) wild type, (B) drp-1(tm1108), (C) eat-3(ad426), and (D) fzo-1(cjn020) animals. Arrows point to inner membrane septae; asterisks to electron-dense inclusions; arrowheads to inclusion bodies; scale bars = 200 nm (JPEG 5098 kb) [file 18_2019_3024_MOESM2_ESM.jpg]

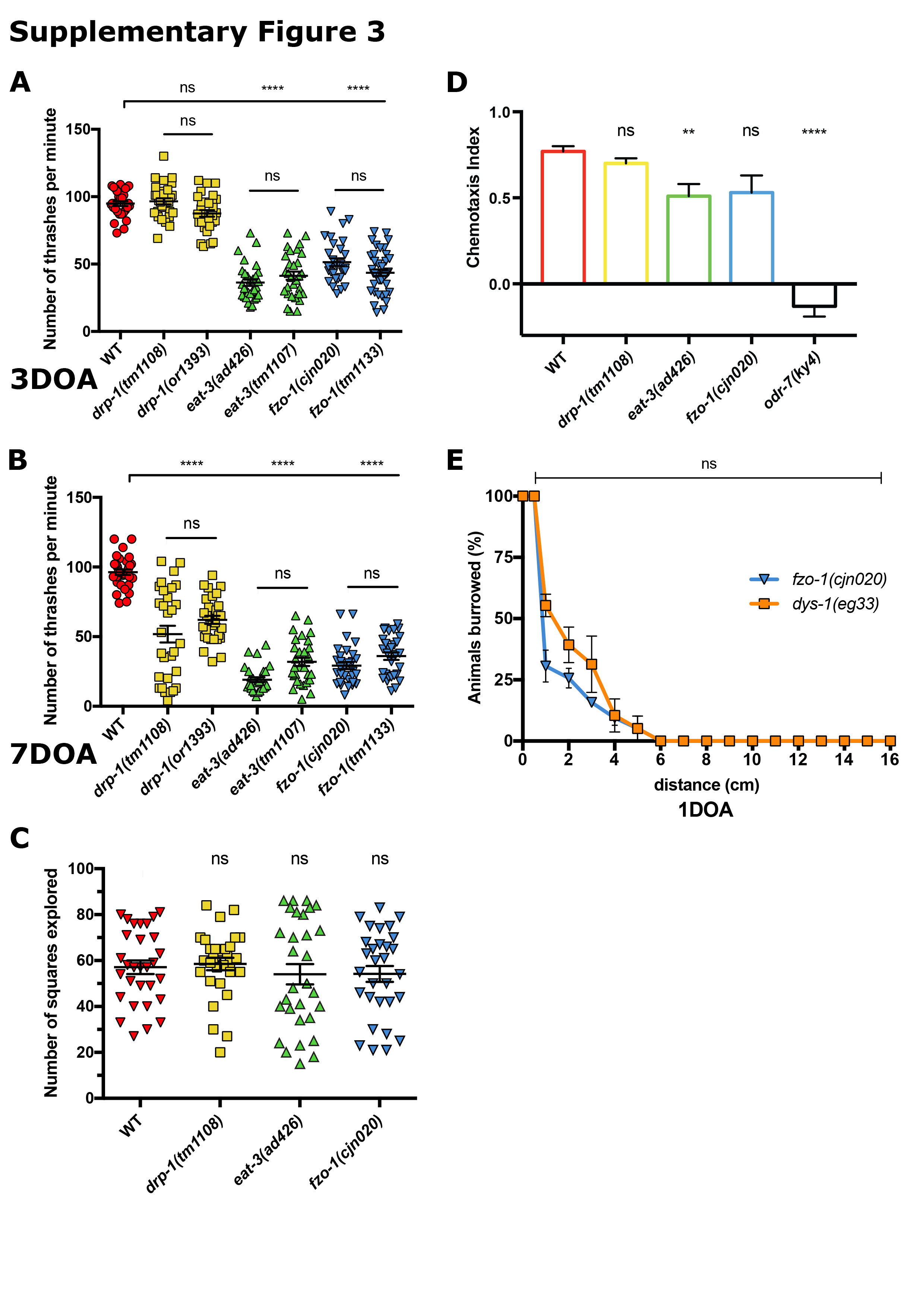

Supplement: Supplementary file 3 — Supplementary Fig. 3. Analysis of additional alleles of mitochondrial dynamics proteins, exploration behaviour, and muscle strength. (A) Quantification of the number of thrashes per minute in liquid. At 3DOA stage both the fusion mutants, fzo-1(cjn020) and eat-3(ad426), and the fission mutant drp-1(tm1108) show no significant difference compared to the relevant secondary allele, fzo-1(tm1133), eat-3(tm1107) and drp-1(or1393). (B) Number of thrashes per minute in liquid at 7DOA stage quantified. Both the fusion mutants, fzo-1(cjn020) and eat-3(ad426) and fission mutant drp-1(tm1108), show no significant difference compared to the relevant secondary allele, fzo-1(tm1133), eat-3(tm1107) and drp-1(or1393). (C) Number of squares entered by each worm after 16 h as a representation of exploration. Symbols show individual animals from three replicate experiments; n ≥ 30 3DOA worms. Data is represented as the mean, ± SEM. (D) Chemotaxis indexes calculated for WT and mutant animals. Chemotaxis indexes were calculated for 1DOA animals exposed to attractant (diacetyl) and control (ethanol) for 1 h. (E) Muscle strength compared to a defective control. fzo-1(cjn020) shows a comparable reduction in muscle strength compared to known muscle defective mutant dys-1. Data is represented as mean cumulative distance covered ± SEM of three replicate experiments. n ≥ 36 worms. ns = P > 0.05, * = P < 0.05, ** = P < 0.01, *** = P < 0.001, **** = P < 0.0001 compared to WT unless indicated otherwise from one-way ANOVA with Dunnett’s post hoc tests for multiple comparisons (JPEG 1134 kb) [file 18_2019_3024_MOESM3_ESM.jpg]

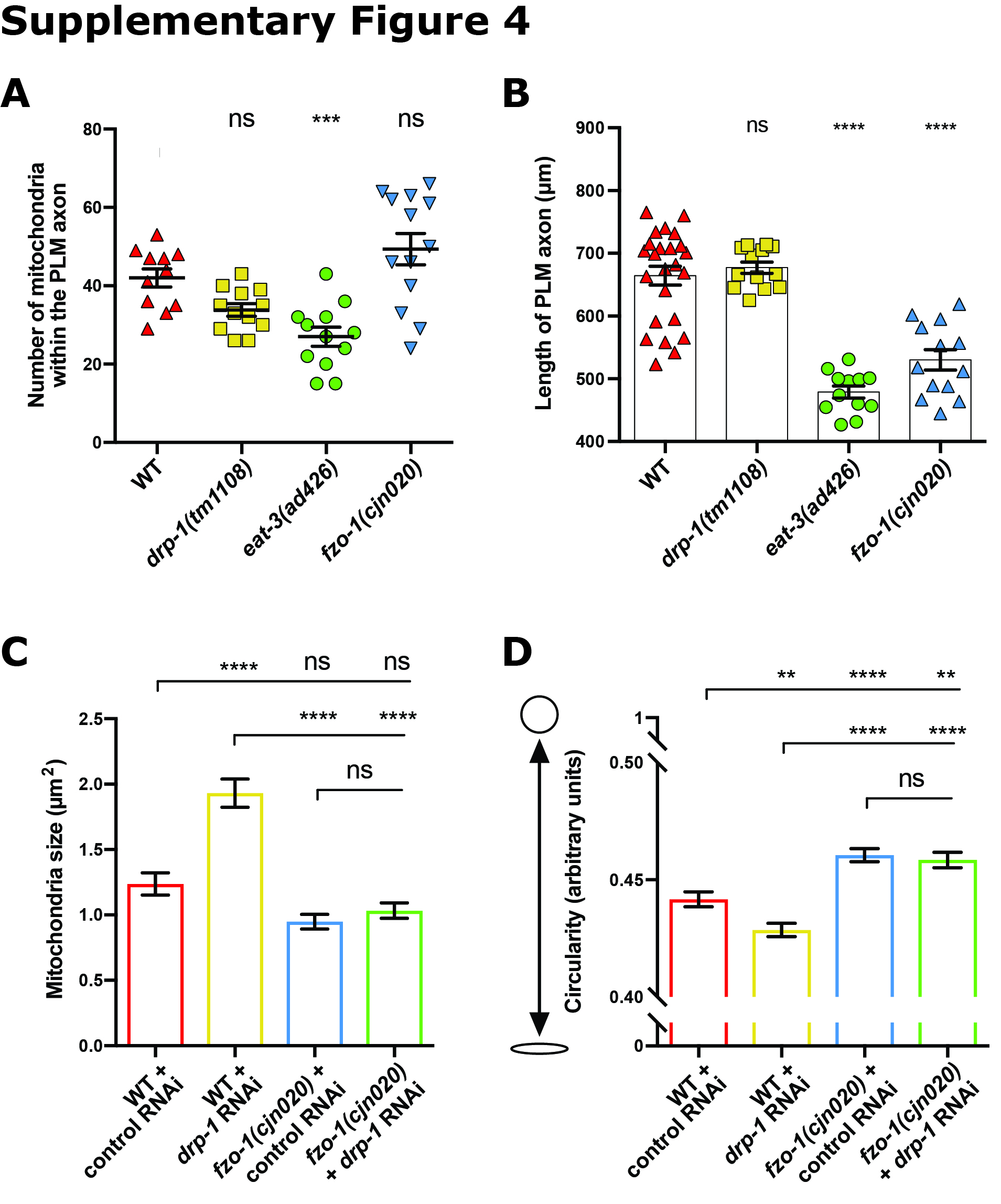

Supplement: Supplementary file 4 — Supplementary Fig. 4. Mitochondrial number and PLM length in the absence of mitochondrial fusion/fission. (A) Number of mitochondria counted per PLM. eat-3 shows a significant reduction in mitochondrial number. n = 12 PLM, 1 per worm. (B) PLM length between fission/fusion mutants. Fusion mutants, fzo-1(cjn020) and eat-3(ad426) both show a reduced PLM length. Symbols show individual PLMs, n = 12 PLM, 1 per worm. Data is represented as the mean, ± SEM. (C) Mean size (µm2) of mitochondria in the body wall muscles as determined using object segmentation (SQUASSH). (D) Mean circularity of mitochondria in the body wall muscles, calculated by fitting each object to a perfect circle and measuring deviation using the following formula (4 x π) x (Area/Perimeter2). A value of 1 represents a perfect circle, and 0 a straight line. ns = P > 0.05, ** = P < 0.01, *** = P < 0.001, **** = P < 0.0001 from one-way ANOVA with Dunnett’s post hoc tests for multiple comparisons (JPEG 2314 kb) [file 18_2019_3024_MOESM4_ESM.jpg]

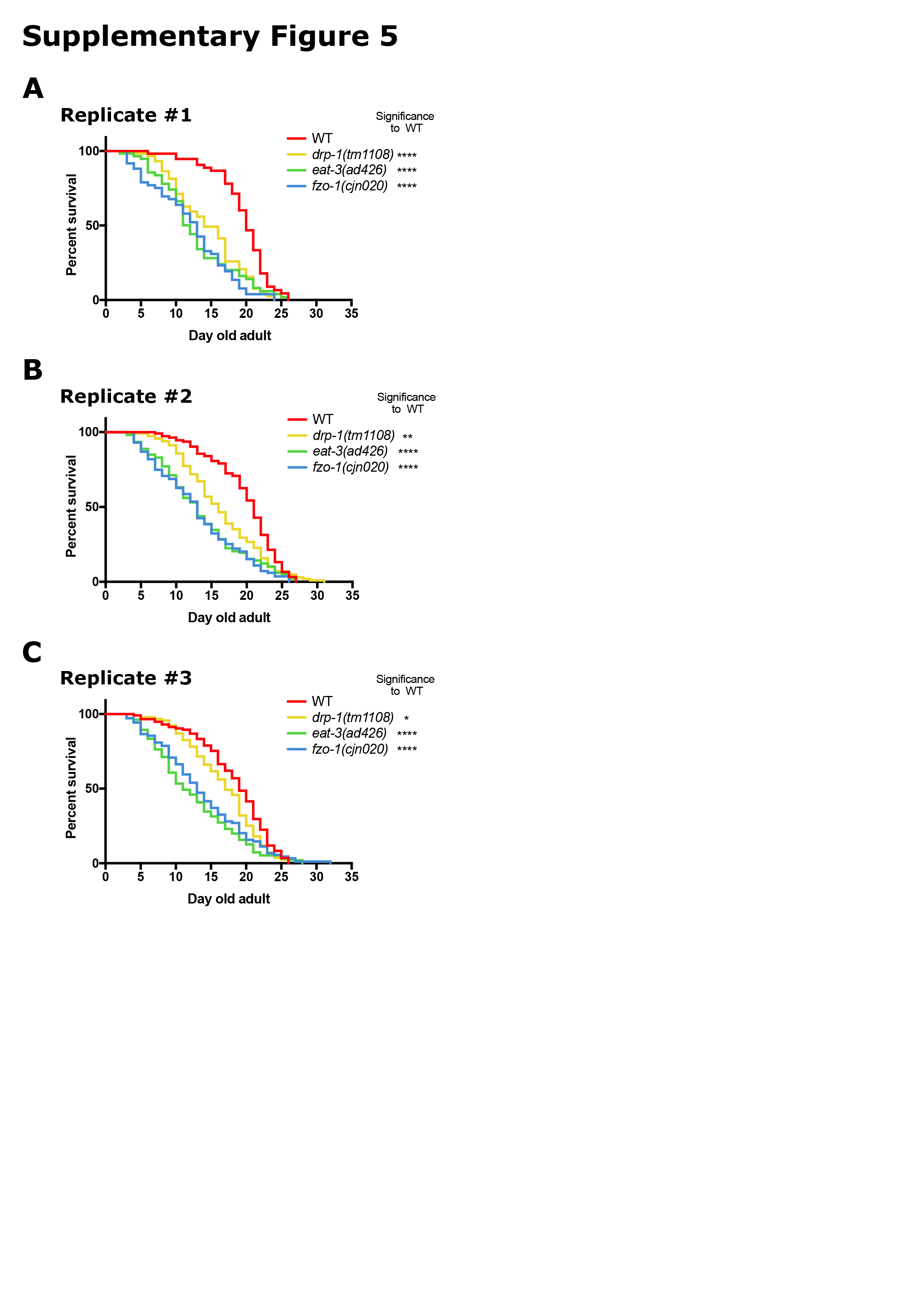

Supplement: Supplementary file 5 — Supplementary Fig. 5. Reduced lifespan in the mitochondrial fusion/fission mutants, single replicate experiments. (A-C) The individual replicate Kaplan–Meier Survival Plots for the mitochondrial fission/fusion mutants. All mutants - drp-1(tm1108), eat-3(ad426) and fzo-1(cjn020) - show significantly reduced mean survival in all three replicates, with maximal survival unchanged. Data is represented as a survival curve of each replicate, ± SEM. * = P < 0.05, ** = P < 0.01, *** = P < 0.001, **** = P < 0.0001 from log-rank (Mantel-Cox) tests to compare the curves; n ≥ 60 worms per genotype for each replicate (JPEG 516 kb) [file 18_2019_3024_MOESM5_ESM.jpg]
