# Supplementary material for: Disruption of mitochondrial dynamics affects behaviour and lifespan in Caenorhabditis elegans
Source: Cell Mol Life Sci. 2019 Mar 6;76(10):1967–85. doi: 10.1007/s00018-019-03024-5 (PMC6478650; doi:10.1007/s00018-019-03024-5)
Supplement: Supplementary file 6 — Supplementary material 6 (DOCX 41 kb) [file 18_2019_3024_MOESM6_ESM.docx]

**Supplementary Table 1.** SQUASSH parameters for segmentation of mitochondria.

| **Parameter** | **Body wall muscle images** | **PLM axon images** |
| --- | --- | --- |
| Objective | 63x | 40x |
| Image resolution (per panel for tilescan) | 2608 x 2608 | 3224 x 3224 |
| Background removal window size | 15 | 20 |
| PSF xy | 0.79 | 0.78 |
| PSF z | 0.71 | 0.68 |
| Regularization | 0.09 | 0.15 |
| Minimum fluorescence intensity of GFP | 0.2 | 0.4 |
