# Supplementary material for: Disruption of mitochondrial dynamics affects behaviour and lifespan in Caenorhabditis elegans
Source: Cell Mol Life Sci. 2019 Mar 6;76(10):1967–85. doi: 10.1007/s00018-019-03024-5 (PMC6478650; doi:10.1007/s00018-019-03024-5)
Supplement: Supplementary file 7 — Supplementary material 7 (DOCX 67 kb) [file 18_2019_3024_MOESM7_ESM.docx]

**Supplementary Table 2.** Strains used in this study.

| **Strain** | **Genotype** | **Origin** |
| --- | --- | --- |
| **SD1347** | *ccIs4251(Pmyo-3::GFP::LacZ::NLS, Pmyo-3::mitochondrial GFP + dpy-20(+))* | CGC |
| **BXN549** | *drp-1(or1393); zdIs5* | This study |
| **BXN592** | *drp-1(tm1108); ccIs4251* | This study |
| **BXN687** | *drp-1(tm1108); eat-3(ad426); ccIs4251, zdIs5* | This study |
| **BXN419** | *drp-1(tm1108); jsIs609; uIs115* | This study |
| **BXN246** | *drp-1(tm1108); zdIs5* | This study |
| **BZ33** | *dys-1(eg33)* | CGC |
| **BXN595** | *eat-3(ad246); ccIs4251* | This study |
| **BXN270** | *eat-3(ad246); jsIs609; uIs115* | This study |
| **BXN251** | *eat-3(ad246); zdIs5* | This study |
| **BXN548** | *eat-3(tm1107); zdIs5* | This study |
| **BXN593** | *fzo-1(cjn020); ccIs4251* | This study |
| **BXN420** | *fzo-1(cjn020); jsIs609; uIs115* | This study |
| **BXN248** | *fzo-1(cjn020); zdIs5* | This study |
| **BXN013** | *fzo-1(tm1133); zdIs5* | This study |
| **BXN038** | *uIs115(Pmec-17::tagRFP); jsIs609(Pmec-4::MLS::GFP)* | This study |
| **QH3135** | *zdIs5(Pmec-4::GFP)* | Massimo Hilliard |
